# Pangenomic Definition of Prokaryotic Species and the Phylogenetic Structure of *Prochlorococcus* spp.

**DOI:** 10.3389/fmicb.2018.00428

**Published:** 2018-03-12

**Authors:** Mikhail A. Moldovan, Mikhail S. Gelfand

**Affiliations:** ^1^A.A.Kharkevich Institute for Information Transmission Problems, Russian Academy of Sciences (RAS), Moscow, Russia; ^2^Faculty of Bioengineering and Bioinformatics, M.V. Lomonosov Moscow State University, Moscow, Russia; ^3^Center for Data-Intensive Biomedicine and Biotechnology, Skolkovo Institute of Science and Technology, Moscow, Russia; ^4^Faculty of Computer Science, Higher School of Economics, Moscow, Russia

**Keywords:** pangenome, prokaryotic species, taxonomy, species definition, monophyly, paraphyly

## Abstract

The pangenome is the collection of all groups of orthologous genes (OGGs) from a set of genomes. We apply the pangenome analysis to propose a definition of prokaryotic species based on identification of lineage-specific gene sets. While being similar to the classical biological definition based on allele flow, it does not rely on DNA similarity levels and does not require analysis of homologous recombination. Hence this definition is relatively objective and independent of arbitrary thresholds. A systematic analysis of 110 accepted species with the largest numbers of sequenced strains yields results largely consistent with the existing nomenclature. However, it has revealed that abundant marine cyanobacteria *Prochlorococcus marinus* should be divided into two species. As a control we have confirmed the paraphyletic origin of *Yersinia pseudotuberculosis* (with embedded, monophyletic *Y. pestis*) and *Burkholderia pseudomallei* (with *B. mallei*). We also demonstrate that by our definition and in accordance with recent studies *Escherichia coli* and *Shigella* spp. are one species.

## Introduction

Numerous definitions of prokaryotic species and methods to divide prokaryotes into distinct, discrete groups have been proposed (Cohan, 2002). Early approaches were based on phenotypic features of prokaryotes, and various phenotypic databases were compiled, e.g., the *Bergey's Manual of Systematic Bacteriology* (Krieg and Holt, 1984) so that each new strain could be characterized based on its phenotype and assigned to a taxonomic group, with a species name assigned after publication in a specialized journal (Funke et al., 1997; Ramasamy et al., 2014; Mohr et al., 2018; Sun et al., 2018). While the number of distinct phenotypic features, such as cell morphology, colony features, biochemical capabilities, pathogenicity, etc. is rather small, and they provide little information about the levels of hierarchy above species (e.g., what similarities are sufficient to merge several species into a genus or several genera into a family), this approach is still widely used by nomenclature communities (Mohr et al., 2018; Sun et al., 2018).

The first formal criterion based on the percentage of DNA–DNA hybridization was proposed by Wayne et al. in 1987 (Wayne et al., 1987). By this definition, organisms with 70% DNA hybridization belong to the same species. As sequencing techniques developed, it became possible to consider phylogenetic trees based on 16S rRNA sequence alignments (Wang et al., 2007), pioneered by Woese et al. (Woese et al., 1985; Woese, 1987). It has been proposed that two organisms with 16S rRNA identity higher than 98% should belong to one species (Cohan, 2002). Further development of this approach relied on whole-genome alignments or average protein identity instead of 16S rRNA (Woese et al., 2000; Thompson et al., 2011; Zhang et al., 2014). Both approaches are not without problems, the most important of which is arbitrariness of the selected thresholds (Clarrige, 2004; Konstantinidis and Tiedje, 2005; Rossi-Tamisier et al., 2015). As a result, some researchers doubt the very existence of prokaryotic species and postulate that the strain is the only biologically meaningful elementary unit of prokaryotic taxonomy (Doolittle and Zhaxybayeva, 2009). The current, integrated definition of bacterial species requires >70% DNA–DNA hybridization, < 5°C ΔTm, < 5% mol G+C difference of total genomic DNA, and >97% 16S rRNA identity (Stackebrandt et al., 2002). However, purely computational, genome-based approaches have been used to suggest reconsidering taxonomy of several groups, e.g., *Synechococcus* (Coutinho et al., 2016).

Recently, another type of approach to the definition of prokaryotic species has been introduced (Bobay and Ochman, 2017). The authors apply the concept of biological species as a reproductively isolated group of lineages to distinguish bacterial species, with homologous recombination taken as an analog of eukaryotic sexual process. The gene (rather, allele) flow is defined as the ratio of horizontally transferred polymorphic sites to vertically transferred polymorphic sites. A species is defined as a group of strains with significantly larger intra-group gene flow compared to the gene flow between the group and any other strain.

Genome sequencing of three *Escherichia coli* strains demonstrated that only 39% of orthologous gene groups (OGGs) contained genes common to all three genomes (Welch et al., 2002). Comparative analyses of these genomes yielded the term *pangenome*, defined as a set of OGGs comprised of all genes from a sample of genomes (Tettelin et al., 2005), not only strains, but also larger taxonomic groups (Snipen and Ussery, 2010) and even all bacteria (Lapierre and Gogarten, 2009). A pangenome can be divided into three OGG categories formed by genes with different degree of presence: (i) *core* OGGs represented in all genomes in the sample; (ii) *shell* OGGs comprised of genes from some considerable fraction of genomes; and (iii) *cloud* OGGs containing genes present in only a minor fraction of genomes (Tettelin et al., 2005; Kettler et al., 2007; Lapierre and Gogarten, 2009; Snipen and Ussery, 2010; Baumdicker et al., 2012; Collins and Higgs, 2012; Gordienko et al., 2013).

**Figure 1:**
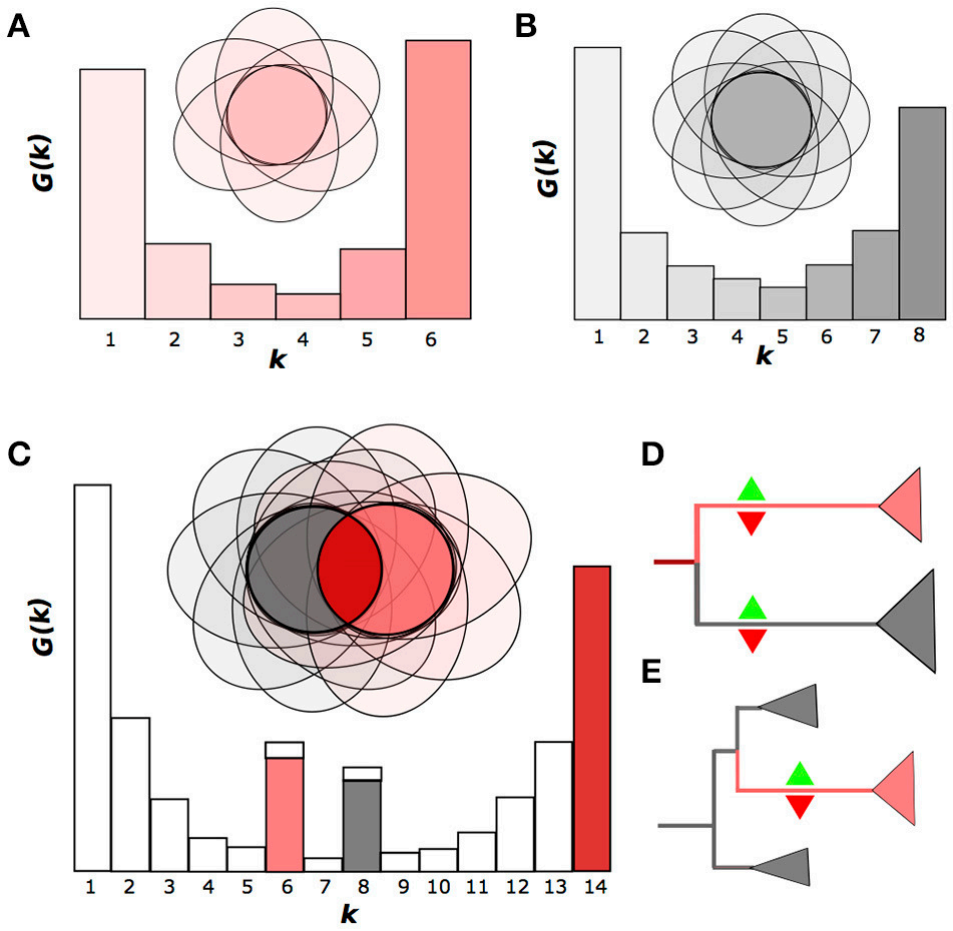
Emergence of nonhomogenous strain sets. **(A,B)**. Two homogenous groups of six and eight strains, respectively, shown as the Venn diagrams, where the ovals represent genomes as sets of genes (OGGs). The gene frequency spectrum function *G*(*k*) is defined as the number of OGGs containing genes from exactly *k* genomes. **(C)** Nonhomogenous group of strains produced by merging two homogenous groups. The two peaks of the *G*(*k*) function correspond to genes specific for homogenous groups. **(D,E)** Two possible scenarios for the emergence nonhomogenous groups: divergence accompanied by independent gene gain and loss in both branches **(D)** or accelerated gene gain and/or loss in an internal clade **(E)**. Green upward and red downward triangles indicate gene gain and loss, respectively.

A convenient way to represent a pangenome is to consider the gene frequency spectrum function *G*(*k*) which if defined as the number of OGGs containing genes from exactly *k* genomes (Baumdicker et al., 2012; Collins and Higgs, 2012) (Figures 1A–C). Typically, if one considers a sample of strains belonging to the same species, the spectrum function of the pangenome is smooth and has a U-like shape with no inner peaks that would be distinct from the noise (Gordienko et al., 2013) (Figures 1A,B). This shape can be obtained in simulations of the gene gain and loss process and shows only a slight dependence on the strain sampling procedure (Collins and Higgs, 2012).

If, however, one has a mixed sample from a small number of species, the spectrum function will have internal peaks (Figures 1C; Gordienko et al., 2013). We will refer to a set of genomes with a U-shaped spectrum function as homogenous, and to a set with internal peaks as non-homogenous. Hence, a homogenous set of strains may not have a subset with many subset-specific genes.

Several papers have discussed bacterial species in the context of phyletic profiles, that is, patterns of gene distribution among strains, and in particular have used the existence of species-specific (more generally, taxon-specific) genes to define species (resp., taxa)(Vitulo et al., 2007; Kahlke et al., 2012). In particular, Kahlke et al. (2012) considered the distribution of branch-specific OGGs when traveling from leaves to the root of the *Vibrionaceae* phylogenetic tree, and demonstrated the existence of spikes at nodes merging branches corresponding to species and higher-level taxa.

Theoretically, there are two basic scenarios for an initially homogenous pangenome of a set of strains to become non-homogenous in course of evolution. (1) If two lineages diverge, neutral evolution or directional selection in both of them would yield independent gene gains and losses, leading to formation of two gene sets, each of which is specific to one of the lineages. This results in formation of two monophyletic, homogenous groups of strains with a number of group-specific genes in both. In the spectrum function, this would yield two peaks at the number of genomes at each group, respectively (Figure 1D). (2) If only one strain in the initial species is affected by strong selection, its descendants would sustain lineage-specific gene gains or losses, that would yield internal peaks in the spectrum function, at the number of genomes from this lineage corresponding to gene gains, and at the number of remaining genomes corresponding to gene losses (Figure 1E). The peaks would form under genetic isolation of the two groups, which means, that the exchange of genes between these groups is limited, similar to the limited allele flow as in the species definition by Bobay and Ochman (2017). Hence the genetic isolation with multiple, independent gene gains and losses provides a natural definition of species that takes into account the accumulation of genomic and, consequently, phenotypic differences in the course of speciation.

Hence, we propose a new procedure for the definition of bacterial species, which is based on the homogeneity of strain sets. In most cases it is consistent with the accepted species structure. According to this *strict* definition, a *monophyletic species* (1) must be monophyletic in a sequence-based tree, (2) should be comprised of a homogenous strain set, and (3) should be the maximal set of strains satisfying conditions 1 and 2. The weak definition requires a species to be either monophyletic or paraphyletic and be a maximal set of strains satisfying condition 2. We have performed a large-scale, two-step search for non-homogenous strain sets among accepted species and applied both versions of our criterion to divide species with non-homogenous pangenomes into distinct groups.

We propose that *Prochlorococcus marinus* is comprised of two species by the strict definition and of three if the weak definition is applied. In addition we show that three species, *Streptococcus equi, Brucella suis*, and *Buchnera aphidicola*, are each comprised of two species by the weak definition. Each partition is robust with respect to strain sampling. As a control, we consider the cases of paraphyletic species *Yersinia pseudotuberculosis* (with *Y. pestis*) and *Burkholderia pseudomallei* (with *B. mallei*), and a monophyletic group *E. coli* and *Shigella* spp., and obtain results consistent with the latest taxonomical studies.

## Results and discussion

### Identification and analysis of non-homogeneous species

We performed a two-step search for non-homogenous species. Firstly, we constructed 110 pangenomes of various species using only 16 strains for each, calculated their spectrum functions *G*(*k*), and then calculated the distribution of the heights of their internal peaks (Supplementary Figure S1). Then we studied the outliers of this distribution in more detail. For that, at the second step, we constructed pangenomes using larger samples and selected species that could be divided into two homogenous strain sets at least one of which had to be monophyletic (see Methods). This yielded the following four species: *P. marinus, S. equi, B. suis*, and *B. aphidicola*.

### *Prochlorococcus marinus* two species in one

*P. marinus* is an ubiquitous, free-living marine photosynthetic cyanobacterium widely used as a model system in marine ecology (Biller et al., 2014). *P. marinus* is abundant in surface waters and dominates phytoplankton biomass being the primary producent in the oligotrophic ocean ecosystem (Biller et al., 2014).

**Figure 2:**
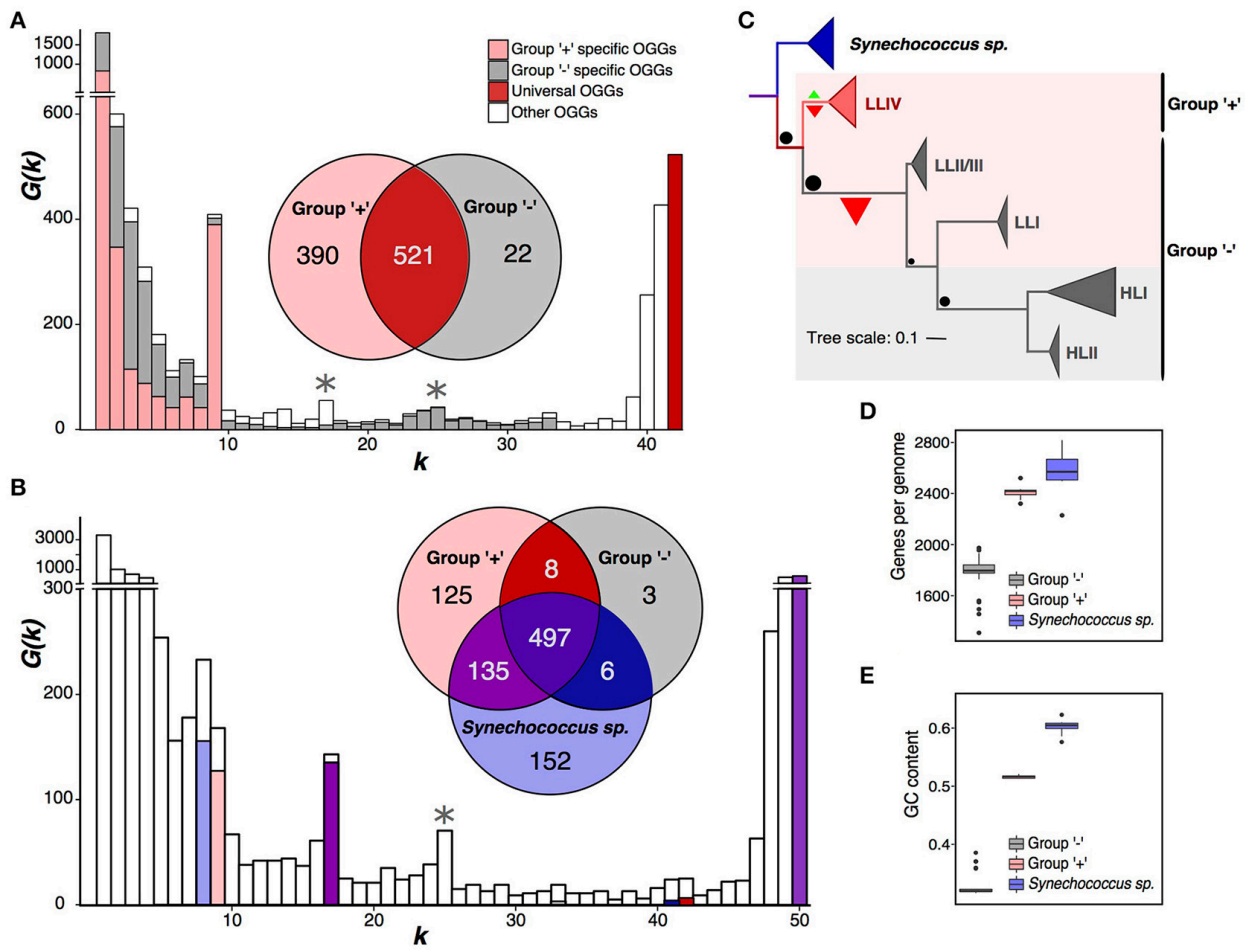
Partition of *Prochlorococcus marinus*. **(A)** *Prochlorococcus marinus* pangenome spectrum function *G*(*k*). Note a large internal peak at *k* = 9. OGGs specific for the plus- and minus-groups are shown in pink and gray, respectively. The Venn diagram shows universal OGGs for each group and their intersections. Asterisks mark additional peaks dividing *P. marinus* into the high-light and low-light groups. **(B)** Same data for the joint pangenome of *P. marinus* and *Synechococcus* spp., the latter shown in blue. **(C)** Phylogenetic tree of *P. marinus* and *Synechococcus* spp. The sizes of the terminal triangles are proportional to the numbers of strains in the respective clades. Green and red triangles mark gain and loss of universal genes, respectively, their size reflects the numbers of gained and lost genes. Black dots size correlates with the number of genes lost in different clades of the *Prochlorococcus marinus* tree. Pink and gray backgrounds reflect partition into the high-light and low-light groups. **(D)** Distributions of the genome size (the number of protein-coding genes). **(E)** Distribution of the GC-content. Outliers in the minus-group distribution are low-light strains.

So far, *P. marinus*, a group of strains considered species by the criterion of rRNA identity >97%, has been divided into five phylogenetically and physiologically distinct clades, which fall into two categories depending on their adaptation to low-light conditions: the monophyletic high-light group (clades HLI and HLII) and the paraphyletic low-light group (clades LLI, LLII/III and LLIV)(Biller et al., 2014) (Figure 2C, Supplementary Figure S3).

The spectrum function of the *P. marinus* pangenome has the highest internal peak (Figure 2A) corresponding to a partition of total 42 strains into two monophyletic sets. One of the sets, containing 9 strains, has 390 group-specific OGGs, and the other set, containing 33 strains, has 22. We call these sets “plus” and “minus,” respectively (Figure 2C, Supplementary Figure S3). The second peak corresponds to the partition into 25 and 17 strains. Together with the first peak, it yields a partition into three groups, one of which matches the plus group and two other are a paraphyletic partition of the minus group. This result does not change if we apply a different E-value threshold to construct OGGs forming the pangenome, and at that the spectrum function *G*(*k*) retains two distinct peaks at same *k* values, which have approximately the same height as those considered here and further (Supplementary Figure S2A) (see Methods).

Thus, by the strict criterion, *P. marinus* is divided into monophyletic plus- and minus-groups. In the terms of the accepted *P. marinus* phylogenetic structure, the plus-group exactly matches the LLIV clade and the minus-group matches the monophyletic group of other clades (HLI, HLII, LLI, and LLII/III). By the weak criterion, the minus-group is further split into the monophyletic high-light clade and the paraphyletic remainder comprised of clades LLI and LLII/III (Figures 2A,C).

An alternative partition of *P. marinus* into 10 species (Thompson et al., 2013), based on sequence features and ecotypes, is only weakly supported by the pangenome analysis, as for most of these species, only few species-specific genes could be identified. Our plus-group corresponds to proposed species *P. swingsii*, whereas other nine species comprise the minus-group. Under the weak criterion, the minus-group is divided into two sets of species: low-light *P. proteus, P. marinus*, and *P. ceticus* and high-light *P. pastoris, P. tetisii, P. neptunis, P. nereus, P. ponticus*, and *P. chisholmii*. Thus, as the five-clade partition, our criterion did not split any of these species and just yielded larger groups comprised of one or several proposed species.

We considered biological functions of OGGs specific for the plus and minus sets (Supplementary Tables S9, S16, S17). Among others, there are: four photosysthem II components, proteins with Fe-S clusters including a cytochrome b6f complex component, DNA reparation enzymes, ion transporters, and signal proteases. Other predicted, subset-specific functions include metabolite transporters, metabolic enzymes, transcription regulators, and cellular division proteins. Hence, subset-specific OGGs might have a considerable impact on the bacterial phenotype.

To test whether these two strain sets actually comprise two separate (sub)species, we considered two spectrum functions *G*(*k*) built on OGGs specific for each set (Figure 2A). The fractions of shell and cloud OGGs were approximately the same in the plus and minus groups. Moreover, the fraction of OGGs common for these groups is smaller than the fraction of group-specific OGGs in their joint pangenome. This further points to the genetical isolation of these groups which resulted in multiple gains and losses of genes with a various degree of presence (core, shell and cloud). Thus, they seem to be divergent with a relatively low level of gene exchange between them.

*Synechococcus* spp. is a large group of marine Cyanobacteria sister to *P. marinus* (Coutinho et al., 2016). To further study the observed separation of the plus- and minus-groups, we considered a joint pangenome of *P. marinus* and a monophyletic clade of eight *Synechococcus* strains as an outgroup (Figures 2B,C, Supplementary Table S1). The spectrum function of this merged pangenome has visible peaks at *k* = 8, 17, and 25. The peak at *k* = 8 reflects *Synechococcus*-specific OGGs. At *k* = 9 we do not observe a distinct peak, but the high *G*(*k*) value reflects the presence of 125 plus-group-specific OGGs. The peak at *k* = 17, reflects 135 OGGs absent in the minus-group, but common for the plus-group and the sampled *Synechococcus* strains. The peak *k* = 25 corresponds to an alternative *P. marinus* strain partition discussed below.

To further show that the plus- and minus-groups should be considered as separate species, we have analyzed numbers of subset-specific genes in the joint pangenome with *Synechococcus*. A comparison of the numbers of OGGs specific for the plus- and minus-groups and for the *Synechococcus* strains (Figure 2B) shows that the numbers of OGGs specific for the plus-group, *Synechococcus* spp., and for the merged set, plus-group+*Synechococcus*, are similar (125, 152, and 135, respectively). The numbers of OGGs specific for the minus-group, all *P. marinus* to the exclusion of *Synechococcus* spp., and the minus-group+*Synechococcus* set are much smaller and again similar (3, 8, and 6 OGGs, respectively). Hence, in the gene content, not only the plus-group and *Synechococcus* spp. differ significantly, but the minus-group differs from the plus-group and from *Synechococcus* spp. approximately to the same degree.

This shows that the small number of OGGs specific to the minus-group results from genome contraction, that has been already shown for *P. marinus* (Kettler et al., 2007; Sun and Blanchard, 2014). The plus-group genomes contain on average 643 protein-coding genes more than the minus-group genomes (Figures 2C,D). Another parameter distinguishing the plus- and minus-group is the GC-content (Kettler et al., 2007; Sun and Blanchard, 2014; Luo et al., 2017) (Figure 2E). The average GC-content differs by about 19%, and the highest GC-content in the minus-group is 13% lower than the lowest GC-content in the plus-group. This result is consistent with observations of decrease in the GC-content accompanying the genome shrinking (Mende et al., 2017; Ríhová et al., 2017).

Thus, several independent observations argue that *P. marinus* is in fact two species: (1) the plus- and minus-groups are monophyletic in the sequence-based tree; (2) there are many OGGs specific for the plus-group and some OGGs specific for the minus-group; (3) in the plus-group and the minus-group pangenomes, the fractions of shell and cloud OGGs are very similar (Figure 2A pink and gray bars), and, moreover, in the integrated pangenome, the fraction of shell OGGs containing genes from both plus- and minus-groups is much smaller than the fractions of group-specific OGGs (Figure 2A white bars). This indicates that gene exchange and parallel gene losses happen within groups much more often than between groups, which leads us to speculate that there exists genetic isolation of the plus- and minus-groups; (4) analysis of the joint pangenome with *Synechococcus spp*. suggests that *Synechococcus* spp and *P. marinus* plus-groups have experienced an approximately similar number of gene gains, whereas the minus-group mainly has been losing genes; (5) the GC-content and the numbers of protein-coding genes in the plus- and minus-groups differ greatly.

The fraction of non-universal genes in a genome is larger for the plus-group than that for the minus-group. Hence, the plus-group pangenome is more diverse than the minus-group pangenome, and the minus-group is likely more prone to genome streamlining than the plus-group (Sun and Blanchard, 2014). This could indicate that the minus-group evolves under stronger selection pressure. This is consistent with lower dN/dS values in the minus-group strains calculated in earlier studies (Sun and Blanchard, 2014; Luo et al., 2017).

Thus, we suggest that *P. marinus* should be viewed as two separate, monophyletic species. If, however, monophyly is not required and the weak criterion is applied, *P. marinus* should be divided into three groups, monophyletic plus-group, monophyletic high-light subset of the minus-group, and paraphyletic low-light subset of the minus-group. Thus, the traditional partition of *P. marinus* strain into the high-light and low-light groups (Rocap et al., 2002; Thompson et al., 2013) is partially supported by the weak criterion. Also, both the weak and strong criteria do not split any clades in previously proposed partitions, yielding combinations of these clades as potential species.

### Weak criterion: three cases of homogeneous, monophyletic branches

**Figure 3:**
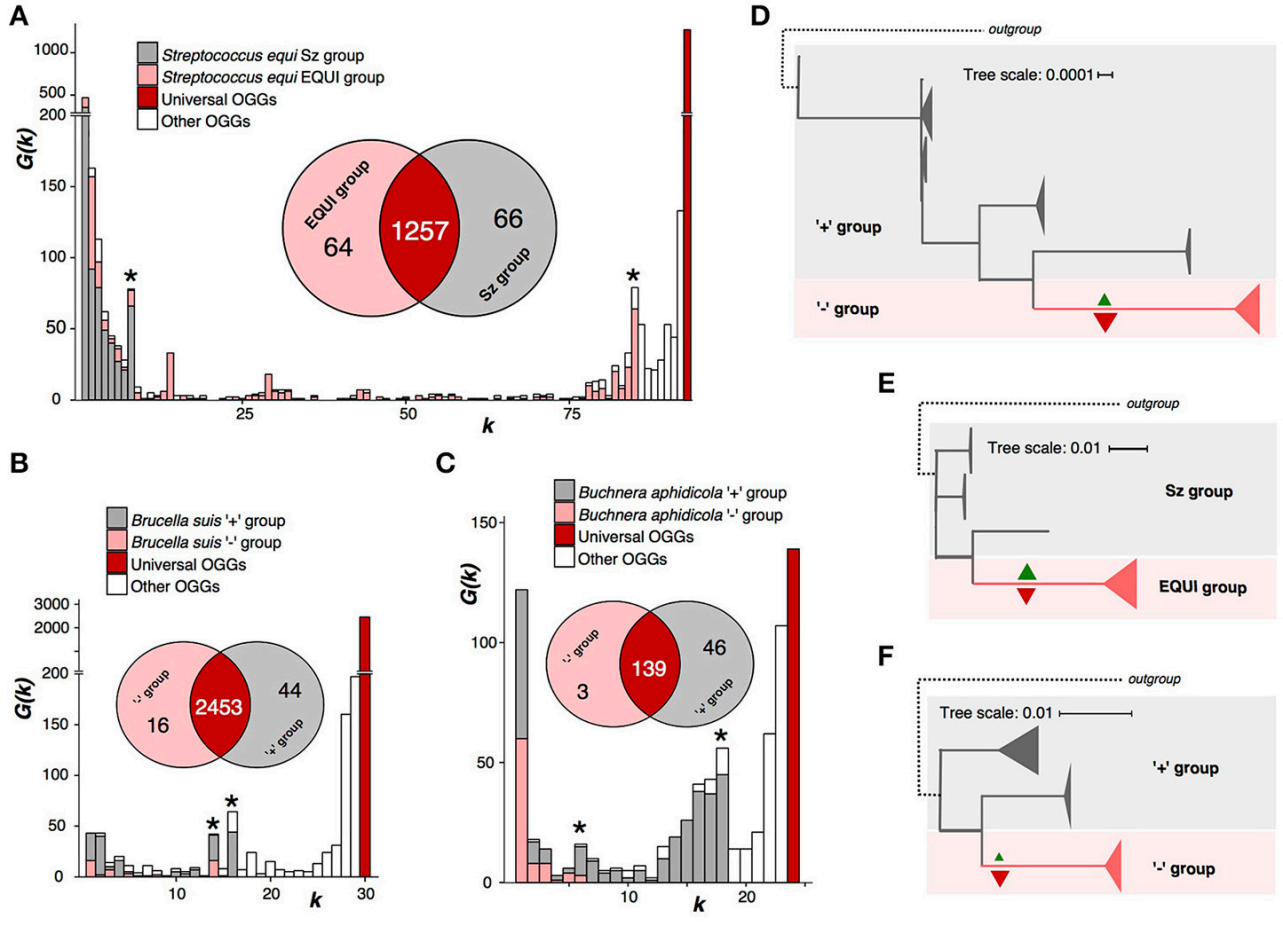
Nonhomogenous species with a monophyletic group and a paraphyletic renainder. Notation as in Figure 2. **(A,D)** *Streptococcus equi*. **(B,E)** *Brucella*. **(C,F)** *Buchnera aphidicola*.

Other species with nonhomogenous pangenomes, *S. equi, B. suis*, and *B. aphidicola*, have strain subsets with homogenous pangenomes, but, unlike the case of *P. marinus*, these species do not satisfy the strict definition. However, they satisfy the weak definition, i.e., in each case there is a monophyletic homogenous group (an internal branch) and a paraphyletic remainder (Figure 3, Supplementary Figures S2B,C,F, S4–S6). All these species seem to be examples of a sudden, lineage-specific change of selection strength and/or direction (Figure 1E), which yields a massive, fast gene loss and gain in this lineage in the context of virtual lack of change in other lineages.

Firmicute bacterium *S. equi* is the pathogen causing the horse disease strangles with high equine mortality rates (Tirosh-Levy et al., 2016). *S. equi* strains are divided into two subspecies, monophyletic *S. equi* subsp. *equi* comprised of pathogenic strains, and paraphyletic *S. equi* subsp. *zooepidemicus*, that are a part of the normal microphlora in horses (Javed et al., 2016) (Figures 3A,E, Supplementary Table S2). The weak criterion partitions *S. equi* into two groups exactly matching the subspecies. The subsp. *equi* has lost 66 genes, among them genes encoding metabolic enzymes, transporters, transcription factors, and CRISPR-associated enzymes; and gained 64 among which are four methylases and three proteins involved in pathogenesis (Supplementary Tables S9, S14, S15).

*B. suis*, a proteobacterium causing infections in animals such as cattle or swine (Ficht, 2010) shows a very similar pattern (Supplementary Table S5). But, unlike *S. equi*, its internal monophyletic clade has lost more genes than it has gained (Figures 3B,D, Supplementary Figure S5). Both gained and lost genes encode enzymes whose functions are important for the bacterial phenotype (Supplementary Tables S9–S11).

*B. aphidicola* is a species of obligate aphid symbionts that supply aphids with essential nutriens (Jiang et al., 2013). The pangenome analysis divides this species into two groups, a monophyletic one that has lost 46 genes, 31 being metabolic enzymes, and gained only three, and a paraphyletic remainder (Figures 3C,F, Supplementary Tables S3, S12, S13). In the phylogenetic trees, branches corresponding to internal, homogenous clades are longer than other branches (Figures 3D–F, Supplementary Figure S5). This could indicate accelerated evolution also on the nucleotide sequence level.

### Control: known, paraphyletic species

To further test our approach, we considered three known cases where some strains in a species traditionally had been grouped into a different species or even a genus due to their medical importance: *Yersinia pestis/pseudotuberculosis, Burkholderia mallei/pseudomallei* and *Shigella* spp./*E. coli* (Supplementary Tables S4, S6, S7) (Liguori et al., 2011; Gordienko et al., 2013; Zimbler et al., 2015).

**Figure 4:**
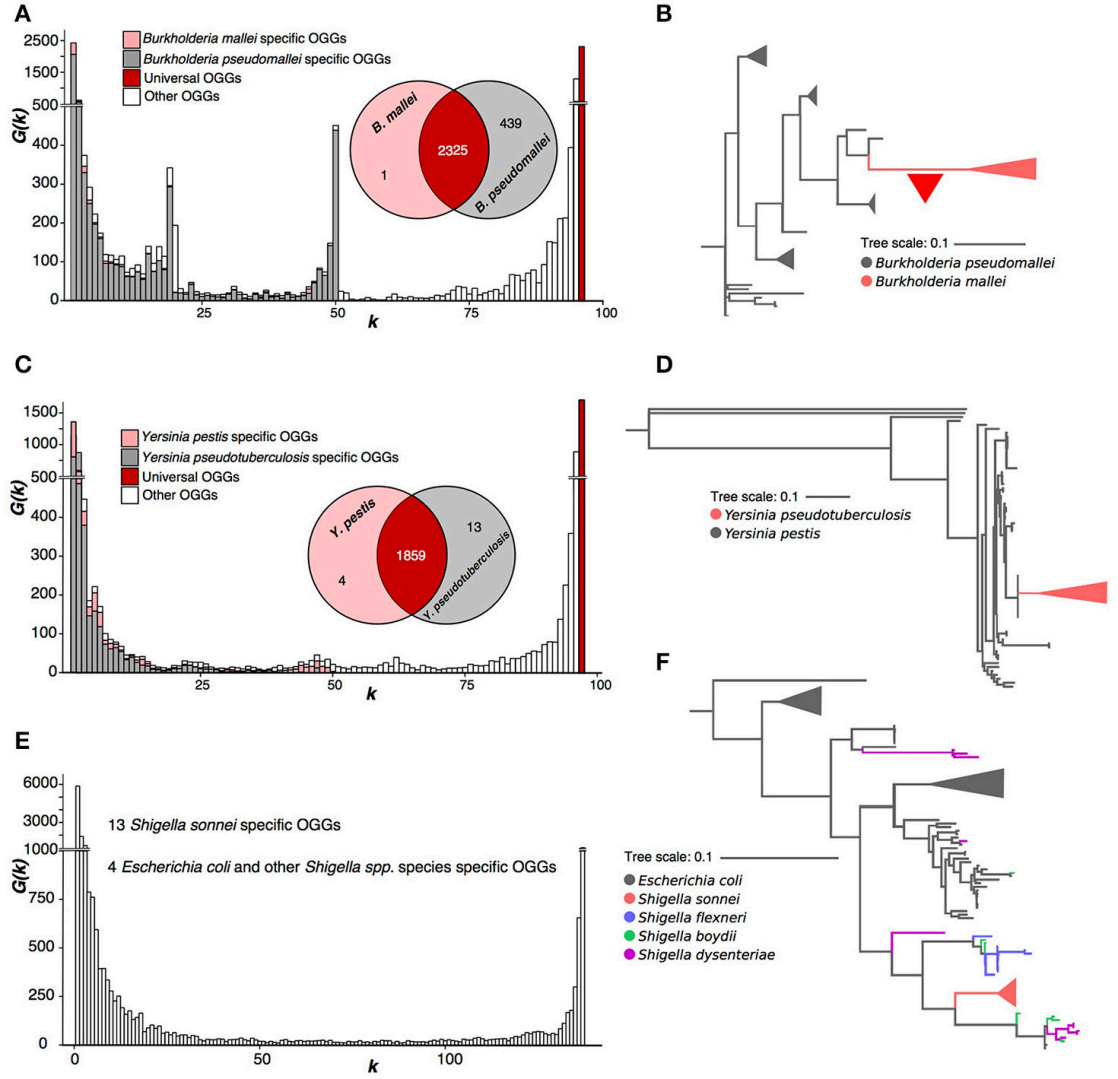
Three paraphyletic species. **(A,B)** *Burkholderia mallei/pseudomallei*. The red triangle represents massive gene loss. **(C,D)** *Yersinia pestis/pseudotuberculosis*. **(E,F)** *Escherichia coli* and *Shigella* spp.

*B. mallei* and *B. pseudomallei* comprise a paraphyletic tree partition, with *B. mallei* forming a separate branch within the *B. pseudomallei* tree (Figure 4A, Supplementary Figure S8), and differ by a large number of OGGs, that is, however, strongly biased with 439 *B. pseudomallei*-specific OGGs and just one *B. mallei*-specific OGG (Figures 4A,B). This indicates intensive gene loss that happened in the recent evolution of the *B. mallei* ancestral lineage. According to the strict criterion, these two species should be merged into one.

*Y. pestis* and *Y. pseudotuberculosis* strains have a homogenous pangenome with only four OGGs specific for *Y. pestis* and 13, for *Y. pseudotuderculosis* (Figure 4C). Similar to the *Burkholderia* case, *Y. pestis* forms a monophyletic branch, while *Y. pseudotuberculosis* is paraphyletic (Figure 4D, Supplementary Figure S7) and again, by the strict criterion these are one species. In this case, the weak criterion yields the same result, as the numbers of species-specific genes do not have distinctive peaks of the spectrum function *G*(*k*).

In both above cases, unlike *P. marinus*, the fraction of OGGs specific for *Y. pestis* and *B. mallei* (Figures 4A,C) is small in the respective pangenomes.

Finally, we considered a joint pangenome of randomly sampled *E. coli* strains and strains belonging to four *Shigella* species, *S. sonnei, S. boydii, S. flexneri*, and *S. dysenteriae*. Only *S. sonnei* had 13 specific OGGs and there were four OGGs absent in *S. sonnei* but present in other *Shigella* spp. and *E. coli* (Figure 4E). In the sequence tree (Figure 4F, Supplementary Figure S9), *S. sonnei* was also the only monophyletic *Shigella* species (z). However, even the weak criterion does not flag out *S. sonnei* as a separate species as, just like in the case of *Yersinia pestis* and *Y. pseudotuberculosis*, the numbers of subset-specific OGGs are too small to produce a distinct peak in the spectrum function.

### Robustness with respect to strain sampling

**Figure 5:**
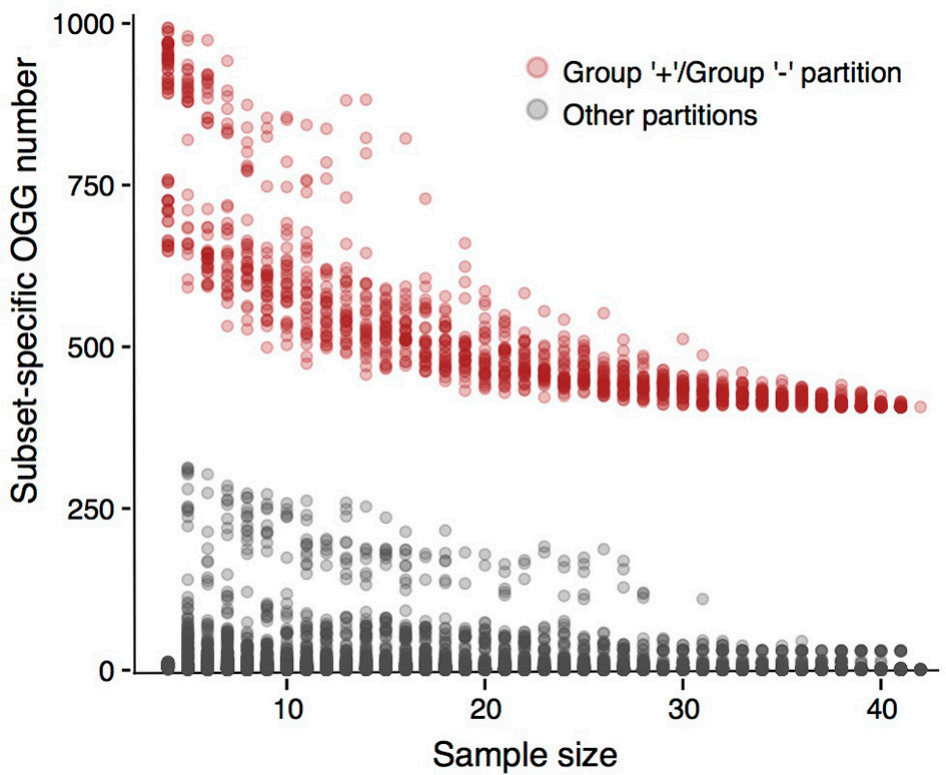
Numbers of subset-specific OGGs for different *Prochlorococcus marinus* partitions. The numbers for partitions consistent with the plus-group/minus-group partition are represented by pink dots, the numbers for other partitions are shown by gray dots. The gray cloud of dots between OGG numbers 100 and 250 corresponds to the high-light/low-light partition.

Next, we tested whether results presented above would depend on strain sampling. To do that, we constructed multiple randomly generated subsamples of various sizes that had to contain strains from all groups formed with our criterion when applied to the initial strain sets (see Methods). We checked whether partitions of these subsets were consistent with partitions of initial sets (Figure 5, Supplementary Figures S10–S13). In the case of *P. marinus*, partitions of all subsets into the plus- and minus-groups yielded much more group-specific genes than any other partition. For the weak criterion, this effect was weaker in the case of the low-light / high light minus-group partition (Supplementary Figure S10). Still, subsamples larger than 15 strains demonstrated the same behavior, as the number of subset-specific genes was larger in the partitions consistent with the initial one than for all other partitions. The same observation held for other partitions generated by the weak criterion: the stable gap between consistent and random partitions appeared in sufficiently large samples, but still smaller than the ones considered in the present study (Supplementary Figures S11–S13). Hence, the presented results are robust.

### Comparison with other methods

We propose here a new approach to the definition of prokaryotic species based on the pangenomic homogeneity, which we define as the absence of strain subsets with numerous subset-specific OGGs. If the subset size is *n*, the definition further requires the remaining *n–k* strains to have more specific OGGs any other subset of *n–k* strains. The required number of subset-specific genes is obtained from the distribution of inner peak heights of the spectrum function *G*(*k*) (Supplementary Figure S1). However, our approach does not necessarily require construction of this distribution, as it has been shown (Kahlke et al., 2012) that inner peaks distinguishable from the noise tend to emerge rather rapidly as subsets of strains diverge from each other (compare Figures 2A, 4E). Thus, our approach is based solely on the genomic content and does not require newly discovered strains to be cultured prior to the taxonomical attribution. This is convenient as most of species cannot be cultured, and hence is an important advantage for environmental microbiology (Turaev and Rattei, 2016). Thus, the only required experimental procedure is metagenomic sequencing followed by the assembly of individual genomes (Turaev and Rattei, 2016).

At present, a disadvantage is the need of a relatively large sample of closely related genomes. Another problem could be sampling bias from uneven taxonomic coverage in genome sequencing, particularly from the presence of almost identical groups of genomes in most databases. As a single strain has a considerable fraction of strain-specific genes (Gordienko et al., 2013), a redundant group of nearly identical strains could yield a peak that could be interpreted as an indicator of a distinct species. However, this situation is easy to identify within the frame of the same approach, as such genomes would have no (or almost none) genome-specific genes, as all such genes would be counted toward the peak. A backup strategy is, of course, genome alignment and analysis of sequence similarity that would be close to 100%.

Another recently proposed arbitrary-threshold free approach to the species determination (Biller et al., 2014) is based on the analysis of the degree of the horizontal gene flow within and between sets of bacterial strains. Two sets of strains are considered separate species if the gene flow between them, manifesting as homologous recombination, is significantly smaller than that within each set. It will be interesting to check whether interruption of the gene flow is a necessary and/or sufficient condition for the formation of the inner peak in the spectrum function *G*(*k*), a preliminary observation being that, the procedure proposed by Bobay and Ochman has yielded a much larger percentage of species that should be split. While our shortlist of species with non-homogenous pangenomes is largely similar to their shortlist of species that should be split into several groups, at the end our criterion is somewhat more consistent with the existing species structure. We suggest that both methods may be applied in conjunction to obtain a decisive solution in specific cases.

## Conclusions

Here, we have performed a systematic analysis of phyletic patterns in bacterial species. This has yielded a new approach to the definition of prokaryotic species based on homogeneity of strain sets. The latter is defined as the absence of subsets with large numbers of subset-universal, specific genes and computationally identified via lack of internal peaks in the spectrum function *G*(*k*). Using the two-step search for non-homogenous bacterial species, we identified four species that could be divided into two groups with numerous group-specific, universal genes. The strict criterion requiring monophyly of both groups retained only one species, *P. marinus*, divided into two groups of strains that we call the plus-group and the minus-group (Figure 2).

The other three species that satisfied the weak criterion seem to have evolved under the scenario of one lineage being affected by a specific mode of selection, yielding lineage-specific gene loss (symbiotic *B. aphidicola*) or a combination of gene loss and gain (*S. equi, B. suis*), resulting in peaks on the *G*(*k*) function. We speculate that such selection regime switches may follow changes of ecological niches. Indeed, the partition of *P. marinus* is largely consistent with both the partition by ecotypes (Thompson et al., 2013) and the accepted partition into five monophyletic clades (Biller et al., 2014). Similarly, the emergence of pathogenicity in *S. equi* subsp. *equi*, the cause of the most prevalent equine infections (Tirosh-Levy et al., 2016), was accompanied by loss of at least three genes responsible for bacterial immunity and 12 genes of general metabolism which accompanied pathogenesis protein acquisition (Supplementary Tables S14, S15).

Our results on paraphyletic composition of *E. coli* (with *Shigella* spp.), *Y. pseudotuberculosis* (with *Y. pestis*) and *B. pseudomallei* (with *B. mallei*) are consistent with recent publications indicating that these species cannot be viewed as monophyletic [*Y. pseudotuberculosis* (Zimbler et al., 2015) and *Burkholderia mallei* (Liguori et al., 2011)] or even are polyphyletic (*Shigella spp*.)(Gordienko et al., 2013).

Our strict criterion is largely consistent with the existing species taxonomy, as it has flagged out only one species among 110 studied ones. An advantage of this approach is that it is based solely on genomic analysis, and hence will become widely applicable as more strains of non-cultured species are sequenced from environmental samples.

The validity of paraphyletic taxa is a subject of debate (Funk and Omland, 2003). Many recognized taxa are paraphyletic (Crisp and Chandler, 1996; Funk and Omland, 2003), e.g., the Vertebrate class Reptilia (Iwabe et al., 2005); the same applies to species (Crisp and Chandler, 1996; Funk and Omland, 2003). Not entering this debate, we introduce two versions of the criterion: the strict one, requiring all groups in the partition to be monophyletic, and the weak one, allowing for a paraphyletic remainder group.

The very biological reality of prokaryotic species is also being debated (Doolittle and Zhaxybayeva, 2009), as the current species definitions strongly depend, on the one hand, on arbitrary thresholds and, on the other, the legacy of tradition. Our approach is somewhat more objective, as it is based on existence of species specific, universal for a given species genes consistent with sequence-based phylogenetic tree.

## Methods

### Identification of candidate non-homogenous species

The following procedure was applied to construct the dataset here, similar to (Moldovan and Gelfand, 2016).

#### Data

One hundred and twenty three collections including ~21000 prokaryotic genomes were downloaded from the Ensembl FTP server (Hubbard et al., 2002) at January 14^th^, 2016.

#### Pangenome dataset

From the initial set of species with sequenced genomes we selected those which had at least 16 annotated strains and at least one strain with a completely assembled genome. Next, we sampled exactly 16 strains for each of the selected species. The sampling was random, but the following conditions had to be satisfied: (1) The numbers of genes in all genomes in a set should differ. (2) The number of genes should be within 3 standard deviations from the average number of genes for the species. (3) The standard deviation of the gene numbers in the selected 16-strain dataset had to be at least ½ of the standard deviation for all genomes of the species.

These conditions defines samples that are both diverse (criterion 3) and do not contain artifacts and outliers resulting from mis-annotations with too small or too large number of genes (criterion 2). This procedure yielded 110 samples (Supplementary Table S18).

#### Construction of pangenomes

For each species, the pangenome construction was based on all-vs.-all protein BLAST (Altschul et al., 1990) for the total set of genes from the sampled 16 strains. At that, the bidirectional best hit (BBH) procedure was implemented to obtain pairs of orthologous proteins, and the mcl algorithm (van Dongen, 2000) was used to construct OGGs; the minimal identity was set to 50%, the minimal E-value, to 10^−10^.

#### Sampling of outliers in the peak height distribution

We calculated the distribution of the highest internal-peak heights in the spectrum function *G*(*k*) (Supplementary Figure S1) over all species, and then selected species corresponding to outliers in this distribution. At the next step, we considered larger samples of strains from the selected species, If the number of annotated strains did not exceed 100, we considered all annotated strains, otherwise we considered 100 randomly sampled strains. The genomes and annotations were downloaded from the NCBI FTP server (Benson et al., 1998) on January 29th, 2017. We then retained four species for further analysis that satisfied the second-peak criterion, see below.

### The second-peak criterion

To determine whether a peak in the spectrum function *G*(*k*) corresponds to a partition of a strain set, we implement the *second-peak criterion*. For a peak at a value k^*^, we consider OGGs contributing to this peak and select the prevalent phyletic pattern, that is, the set of strains most frequently forming these OGGs. We then consider whether a symmetric peak is formed at *n*–k^*^. The second-peak criterion is satisfied if this peak exists, and if it is mainly formed by OGGs having a complementary phyletic pattern. This corresponds to gene gains (main peak) and gene losses (the second peak) in the selected k^*^ strains or, conversely, to gene losses and gains, respectively, in the remaining strains. These alternatives may be resolved by analysis of outgroups, but it is not necessary in the context of this study.

### Pangenome construction

Orthologous gene groups (OGGs) forming pangenomes were constructed using ProteinOrtho (Lechner et al., 2011) on annotated protein sequences. The sensitivity was controlled with BLAST (Altschul et al., 1990) E-value thresholds. Two E-value thresholds, 10^−10^ (Figures 2–4) and 10^−25^ (Supplementary Figure S2), were used, yielding consistent results.

### Robustness analysis

As sampling biases could potentially introduce noise to our data, we re-analyzed the considered species using multiple random sampling for pangenomes built with the E-value threshold of 10^−10^. For each species, we considered subsamples of strains of various sizes. If the number of considered species *n* was less than 50, we analyzed 30 randomly generated strain subsets for each sample size *n*′ starting with four strains and ending with all strains considered in the present study. For species with the number of strains exceeding 50 we analyzed 10 such subsets. Each subset had to include at least two strains from each of the groups proposed in the present study and all subsets had to differ. In the pangenomes of the constructed subsets we compared the numbers of specific OGGs supporting partitions consistent with the proposed partition into k^*^ and *n*′-k^*^ strains and non-zero numbers of specific OGGs supporting other partitions into k^*^ and *n*′-k^*^ strains. We considered the partition to be robust if with the increase of *n*′ the range of subset-specific OGG numbers decreased and the distance between two clusters and subset-specific OGG numbers became constant.

### Phylogenetic trees

Phylogenetic trees of species were constructed with the maximum likelihood method (Felsenstein, 1973) implemented in the PhyML package (Guindon and Gascuel, 2003), using concatenated alignments of nucleotide sequences of one hundred genes present in all strains and in outgroups, with 20 bootstraps. The trees were rooted by closely related, outgroup species. Trees were visualized with EvolView (Zhang et al., 2012) and ItoL (Letunic and Bork, 2016) web resources. Outgroups were selected using the tree obtained from the MicrobesOnline (Dehal et al., 2010) web server as representatives of closely related species (Supplementary Table S8).

### Additional methods

Multiple alignments were constructed using Muscle (Edgar, 2004). GO-terms were assigned to OGGs using InterPROscan (Zdobnov and Apweiler, 2001). Data were visualized with R package ggplot2. Custom scripts were written in python 2.7 and are available online at “https://github.com/mikemoldovan/pangenomes_and_species”.

## Author contributions

MM and MG: designed the research; MM: wrote programs and performed calculations; MM and MG: interpreted the results, wrote the paper and approved the submitted version.

### Conflict of interest statement

The authors declare that the research was conducted in the absence of any commercial or financial relationships that could be construed as a potential conflict of interest.
