# Supplementary material for: Pangenomic Definition of Prokaryotic Species and the Phylogenetic Structure of Prochlorococcus spp
Source: Front Microbiol. 2018 Mar 12;9:428. doi: 10.3389/fmicb.2018.00428 (PMC5857598; doi:10.3389/fmicb.2018.00428)
Supplement: Supplementary file 1 [file DataSheet1.PDF]

Supplementary Material

Pangenomes and the definition of prokaryotic species

Mikhail A. Moldovan*, Mikhail S. Gelfand

*** Correspondence:** Mikhail A. Moldovan: mika.moldovan@gmail.com

1 Supplementary Figures and Tables

1.1 Supplementary Figures

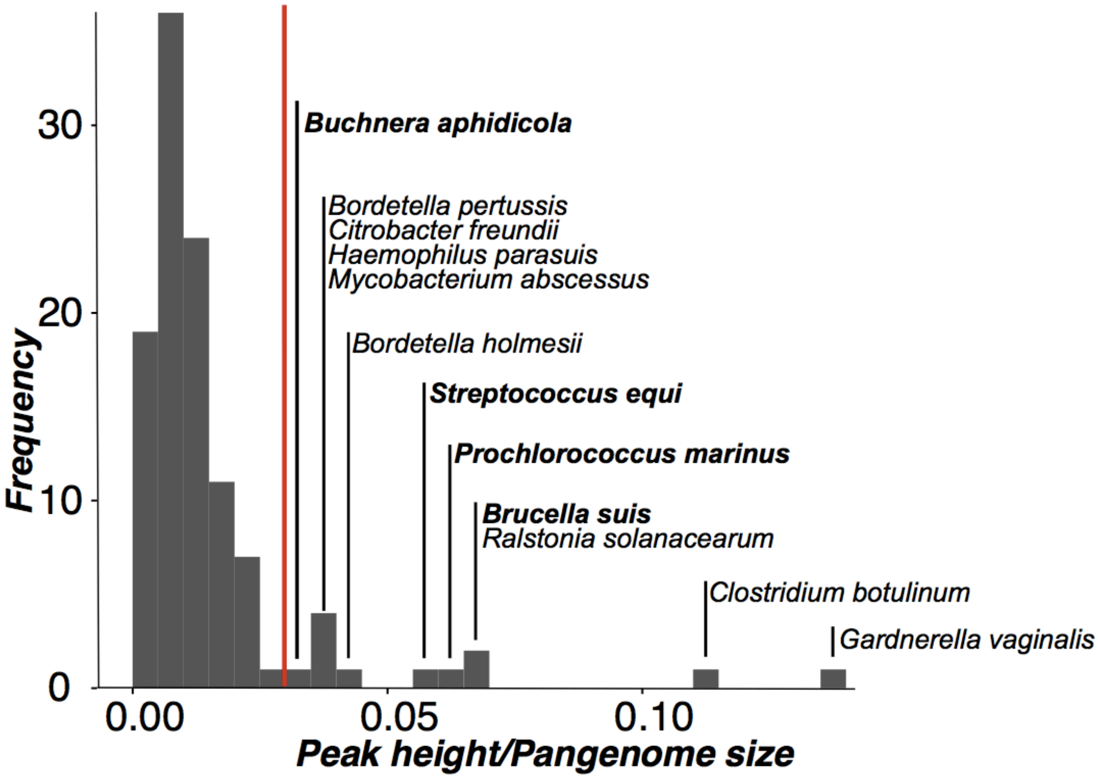

Supplementary Figure S1 | Distribution of the relative peak heights in 110 initially considered pangenomes. The red line marks the used threshold. Bold font marks species satisfying the second peak criterion and hence considered in subsequent analyses.

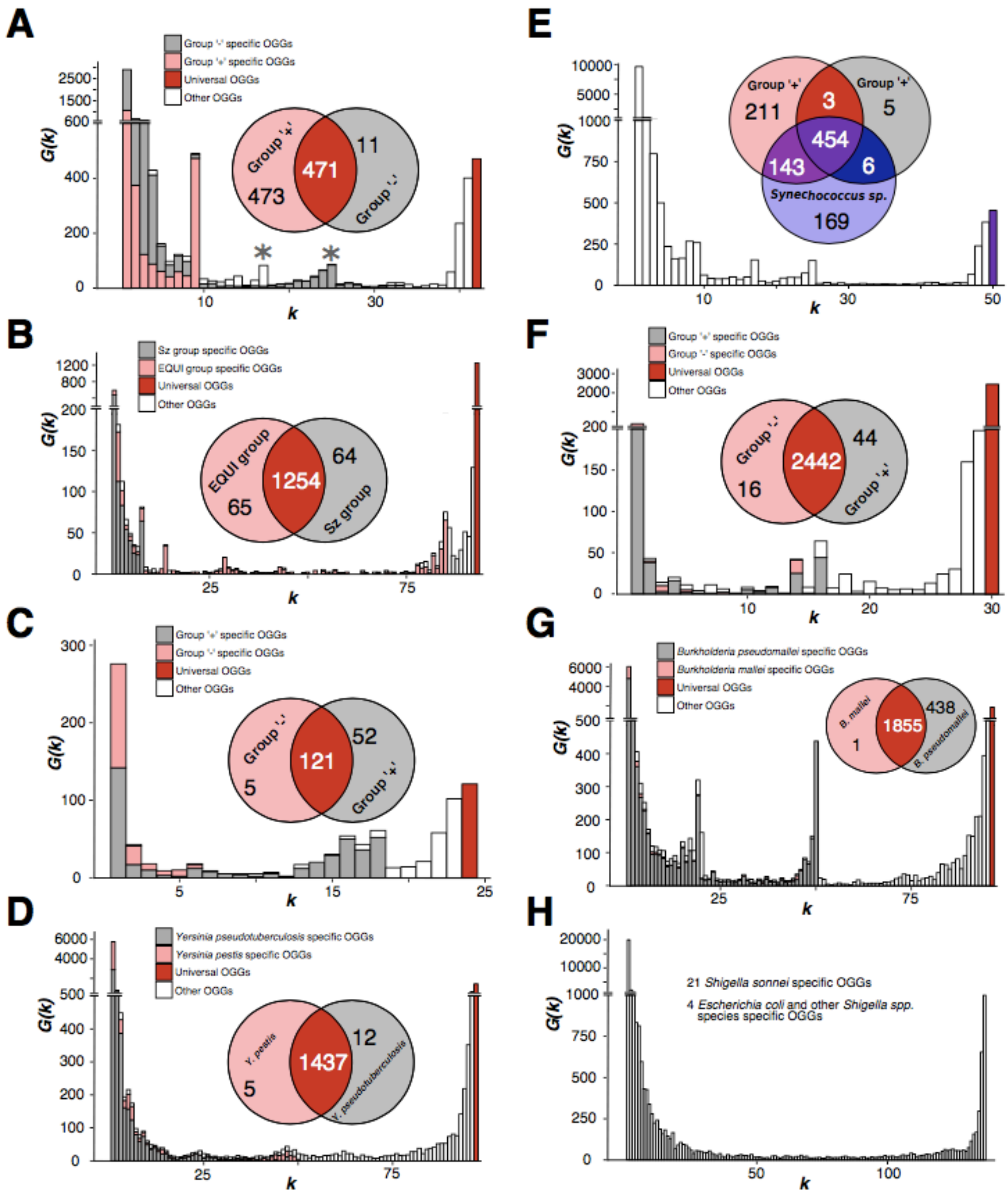

Supplementary Figure S2 | The $G(k)$ functions and the numbers of group-specific OGG built with the E-value threshold 10^{-25}. a. *Prochlorococcus marinus*. Asterisks (*) mark peaks corresponding to the high-light / low-light partition. **b**. *Streptococcus equi*. **c**. *Buchnera aphidicola*. **d**. *Yersinia pestis*and *Y. Pseudotuberculosis*. **e**. *Prochlorococcus marinus* and *Synechococcus sp.* **f**. *Brucella suis*. **g**. *Burkholderia mallei* and *B. Pseudomallei*. **h**. *Escherichia coli* and *Shigella spp*. Horizontal axis — number of strains, vertical axis — number of OGGs present in exactly that number of strains.

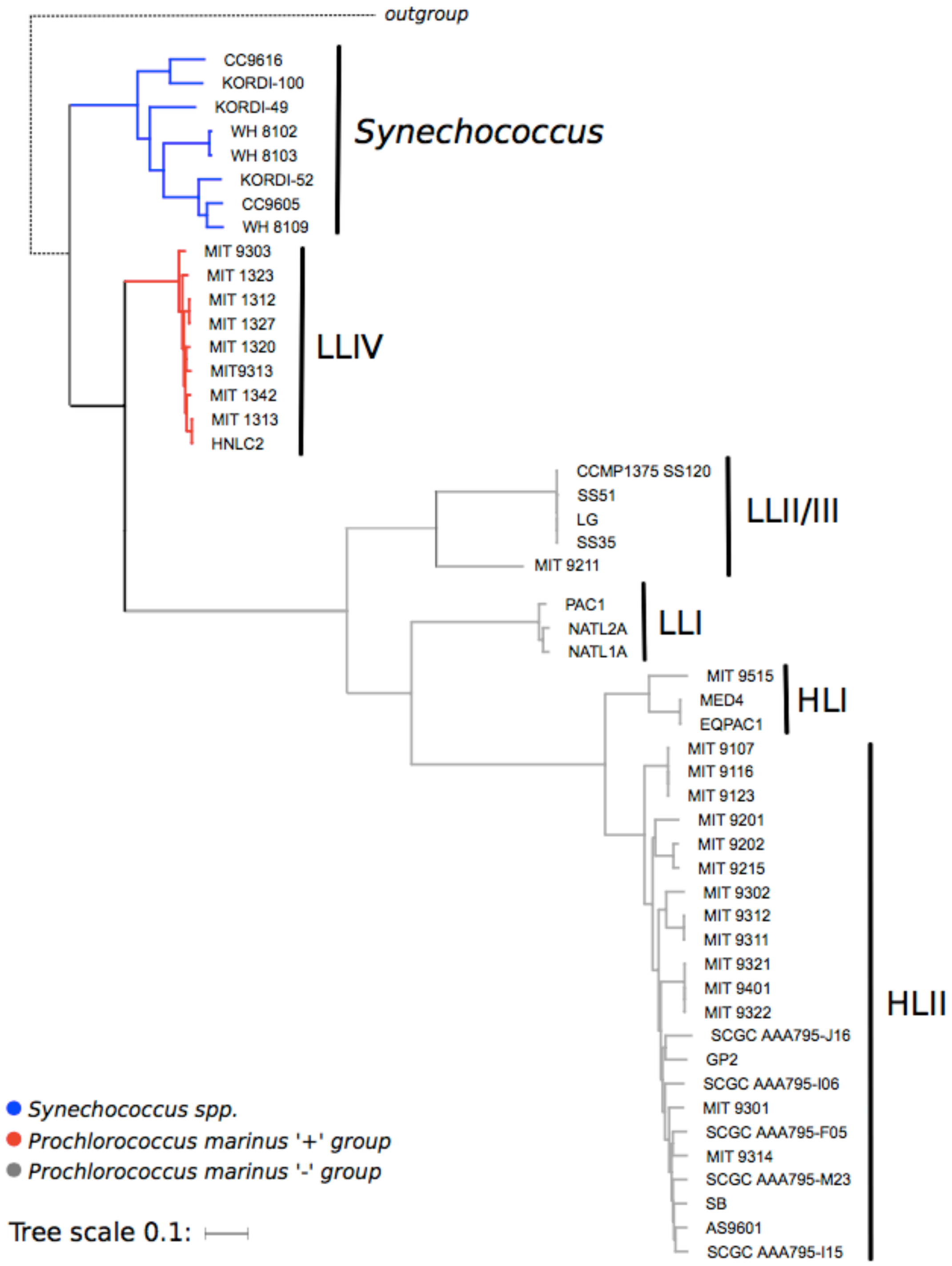

Supplementary Figure S3 | Phylogenetic tree of *Prochlorococcus marinus* and *Synechococcus* spp.

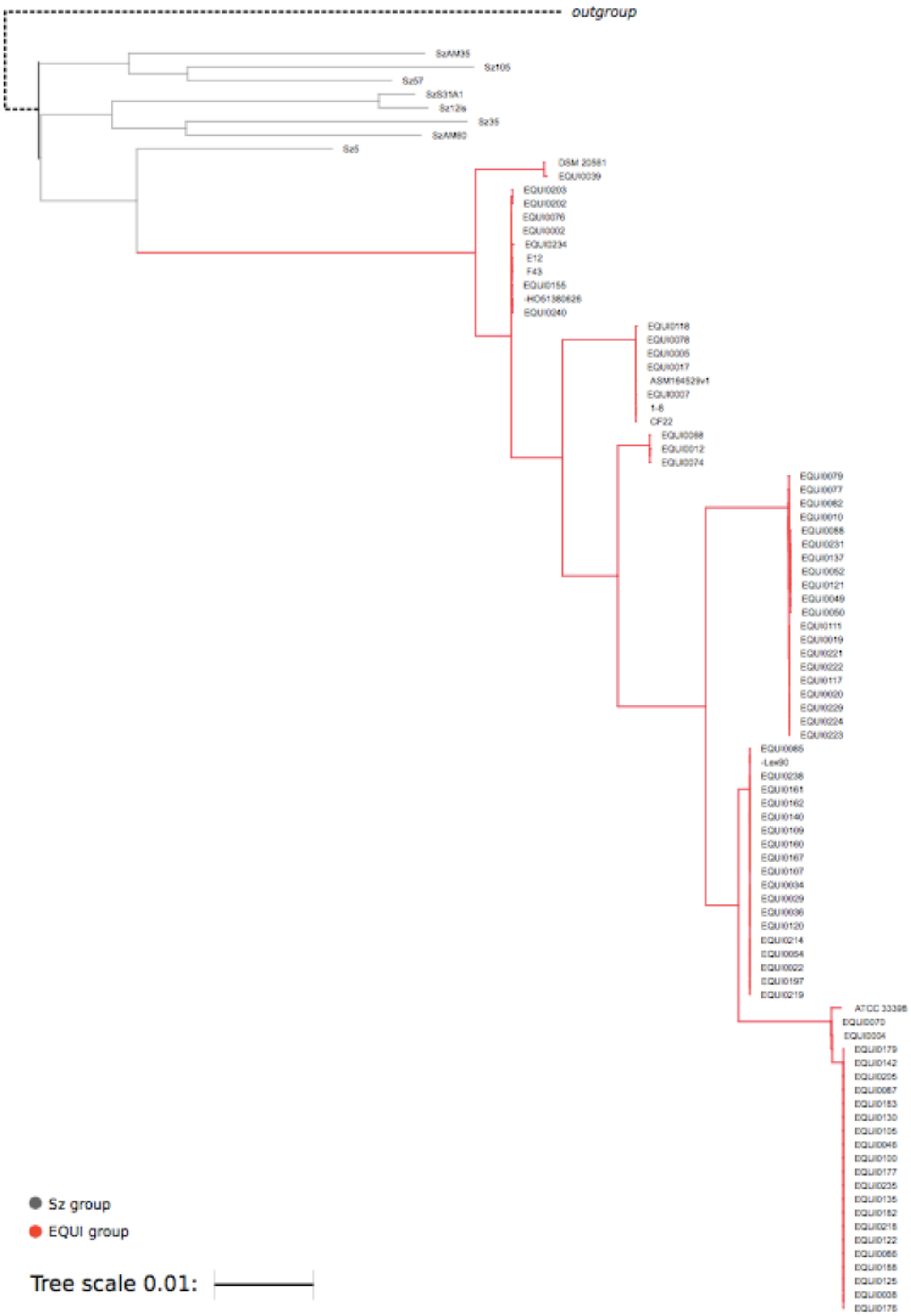

Supplementary Figure S4 | Phylogenetic tree of *Streptococcus equi*.

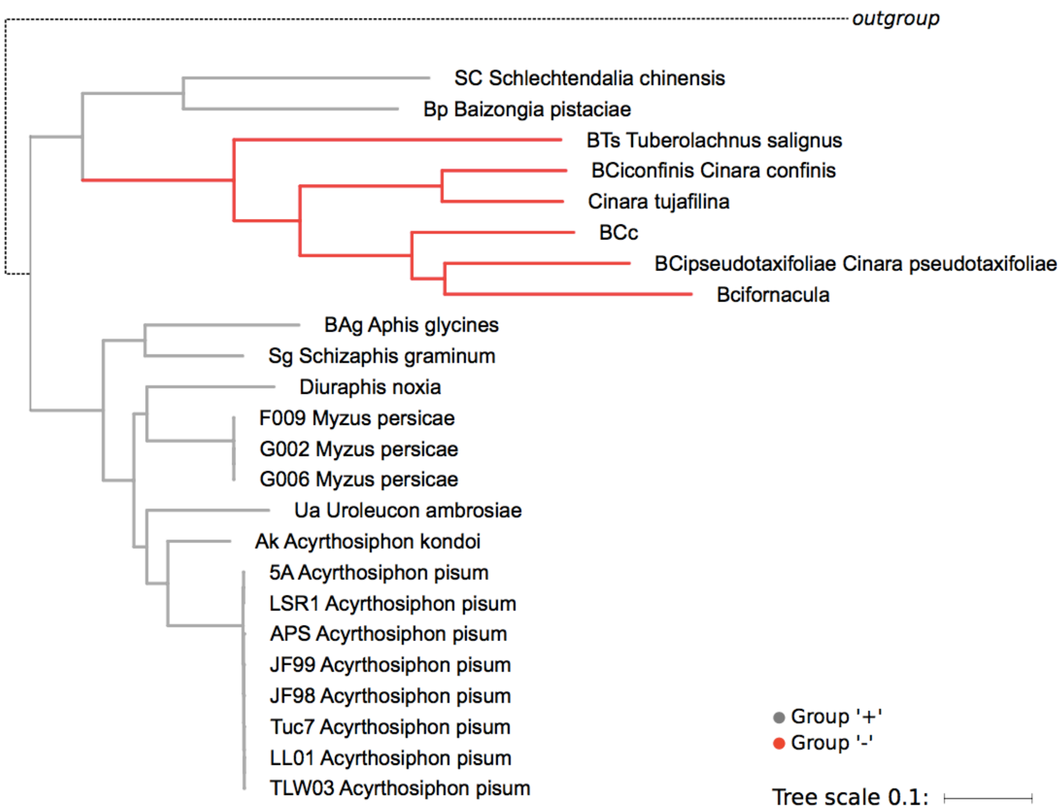

Supplementary Figure S5 | Phylogenetic tree of *Buchnera aphidicola*.

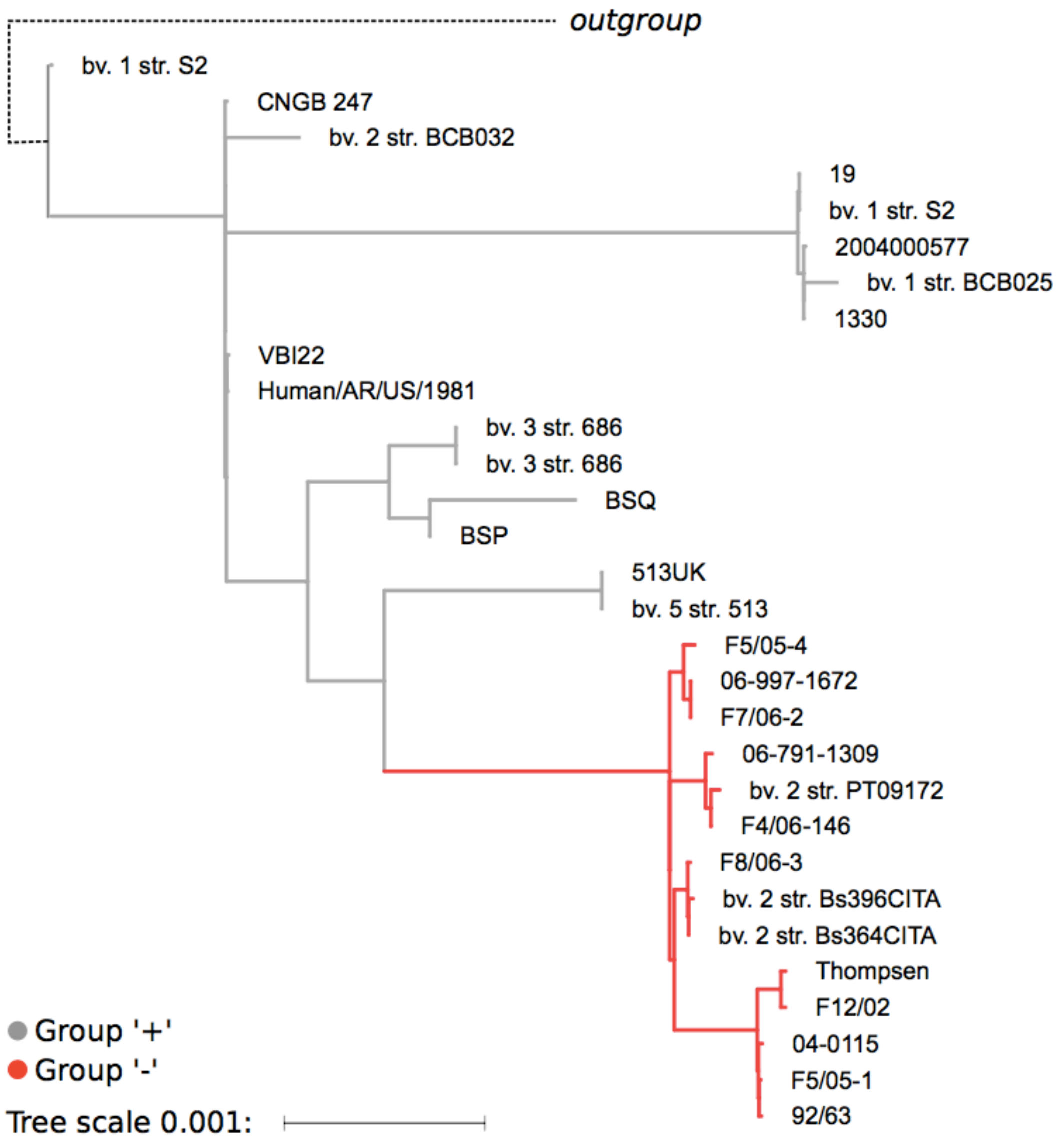

Supplementary Figure S6 | Phylogenetic tree of *Brucella suis*.

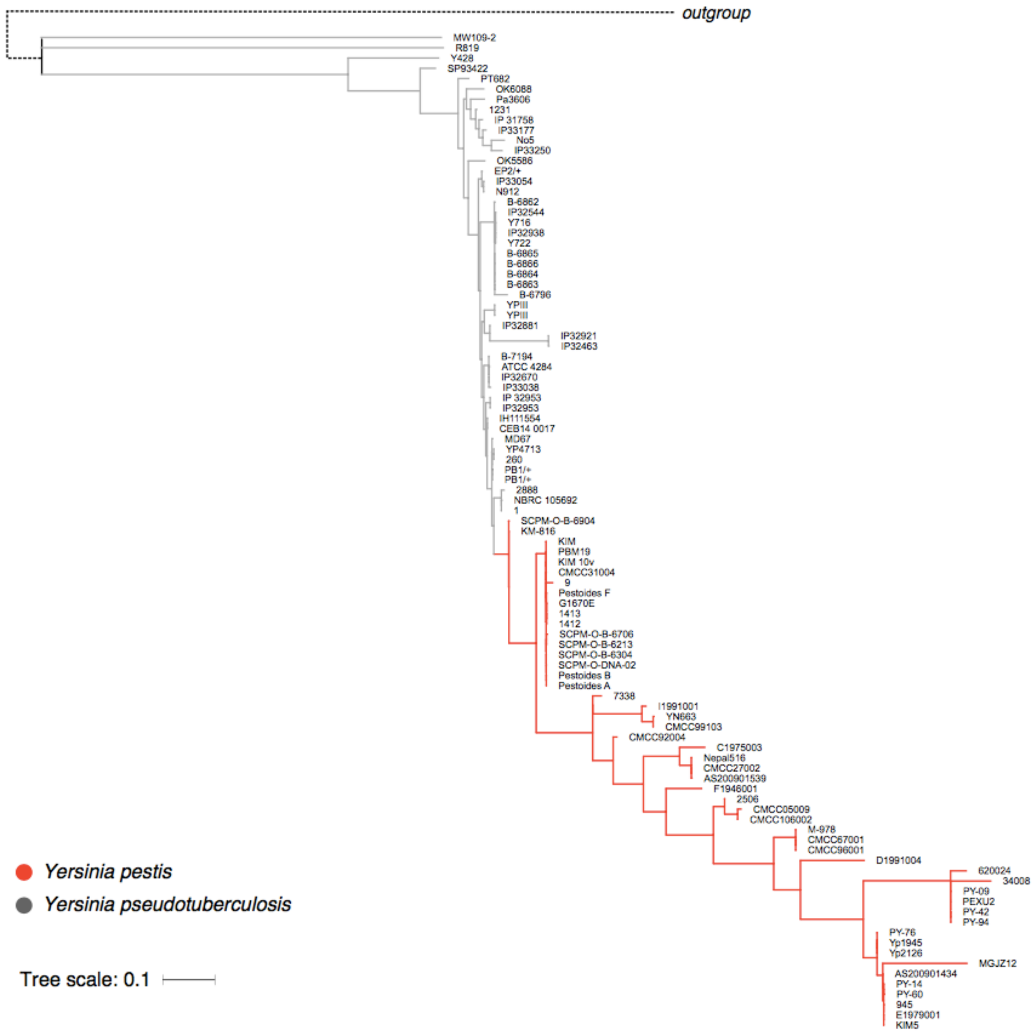

Supplementary Figure S7 | Phylogenetic tree *of Yersinia pestis* and *Y. pseudotuberculosis*.

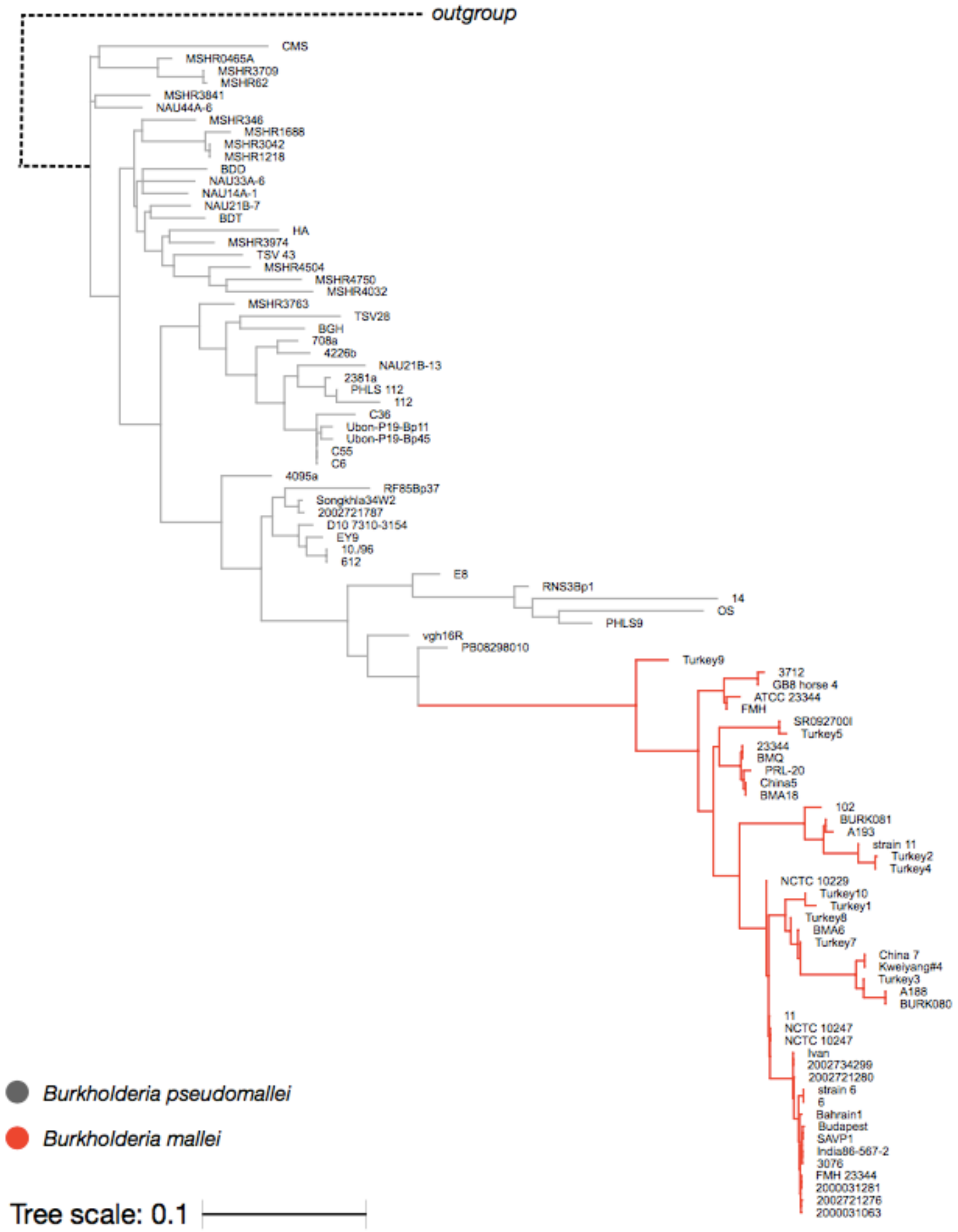

Supplementary Figure S8 Phylogenetic tree of| *Burkholderia mallei* and *B. pseudomallei*.

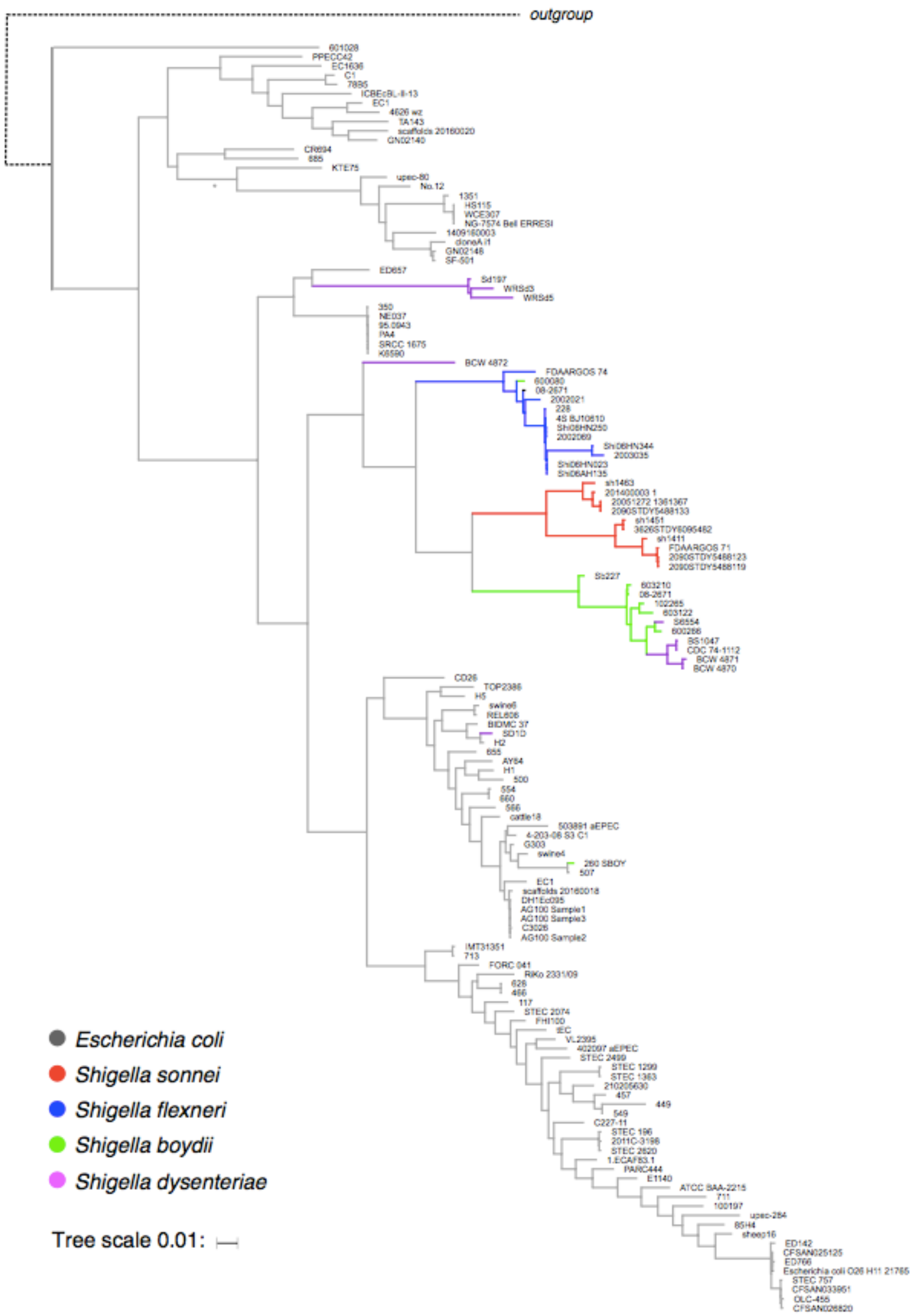

Supplementary Figure S9 | Phylogenetic tree of *Escherichia coli* and *Shigella* spp.

e

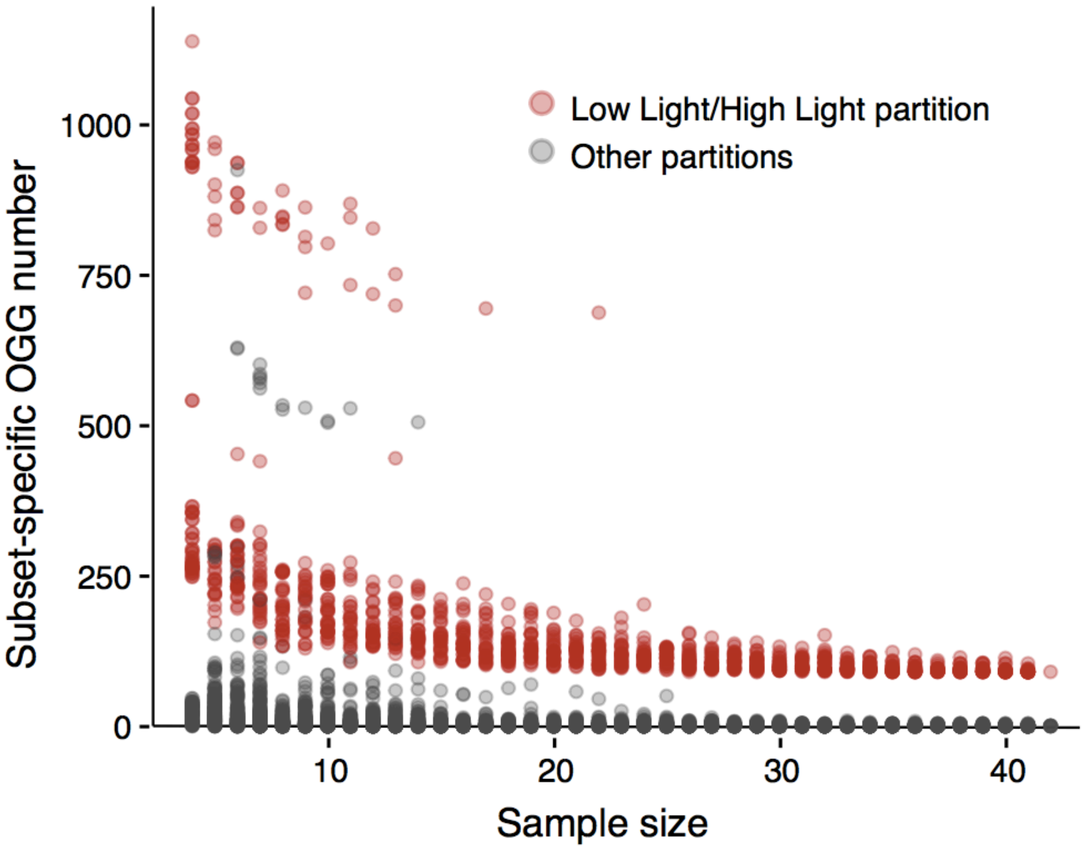

Supplementary Figure S10 | Numbers of subset-specific OGG for different *Prochlorococcus marinus* partitions. The numbers for partitions consistent with the low-light/high-light partition are represented by pink dots, the numbers for other partitions are shown by grey dots. The cloud above the two main clusters represents partitions consistent with the plus-group/minus-group partition.

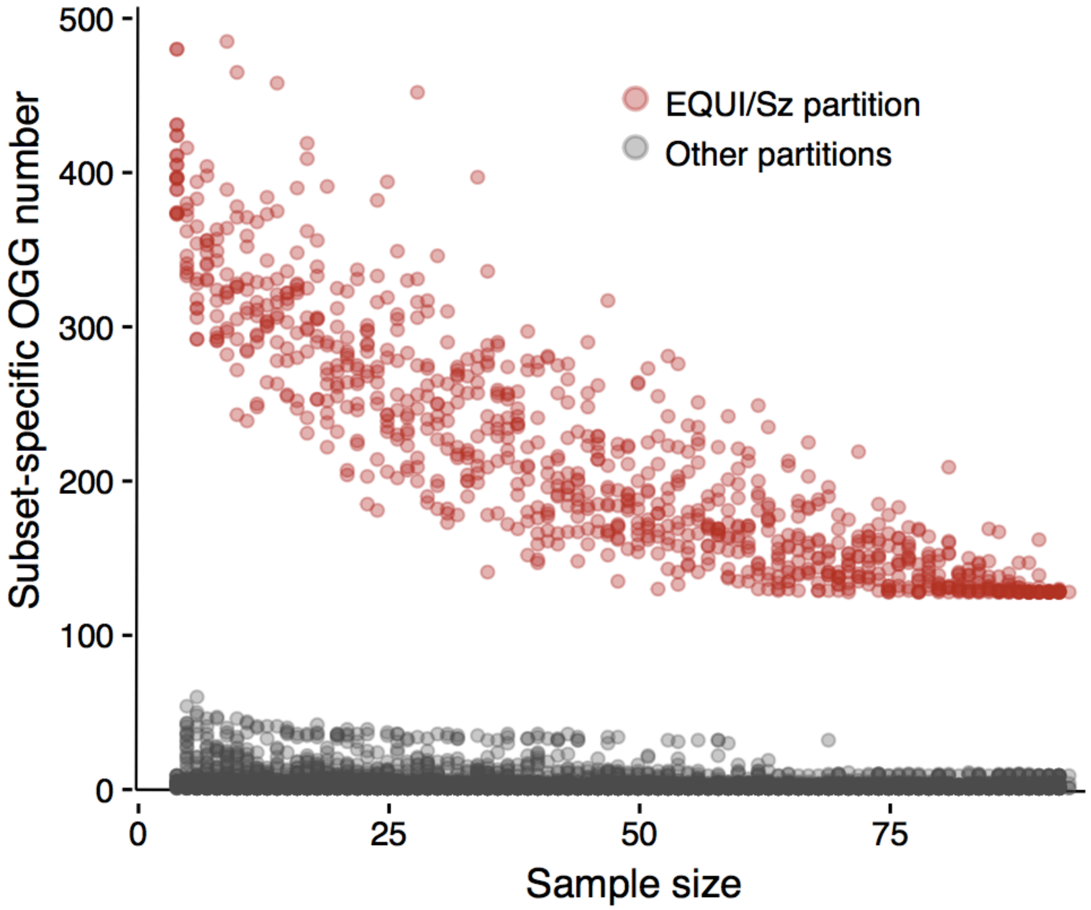

Supplementary Figure S11 | Numbers of subset-specific OGG for different *Streptococcus equi* partitions. Subset-specific OGG numbers for partitions consistent with the EQUI-Sz (subsp. *equi* / subsp. *zooepidemicus*) partition are represented by pink dots, the numbers for other partitions are shown by grey dots.

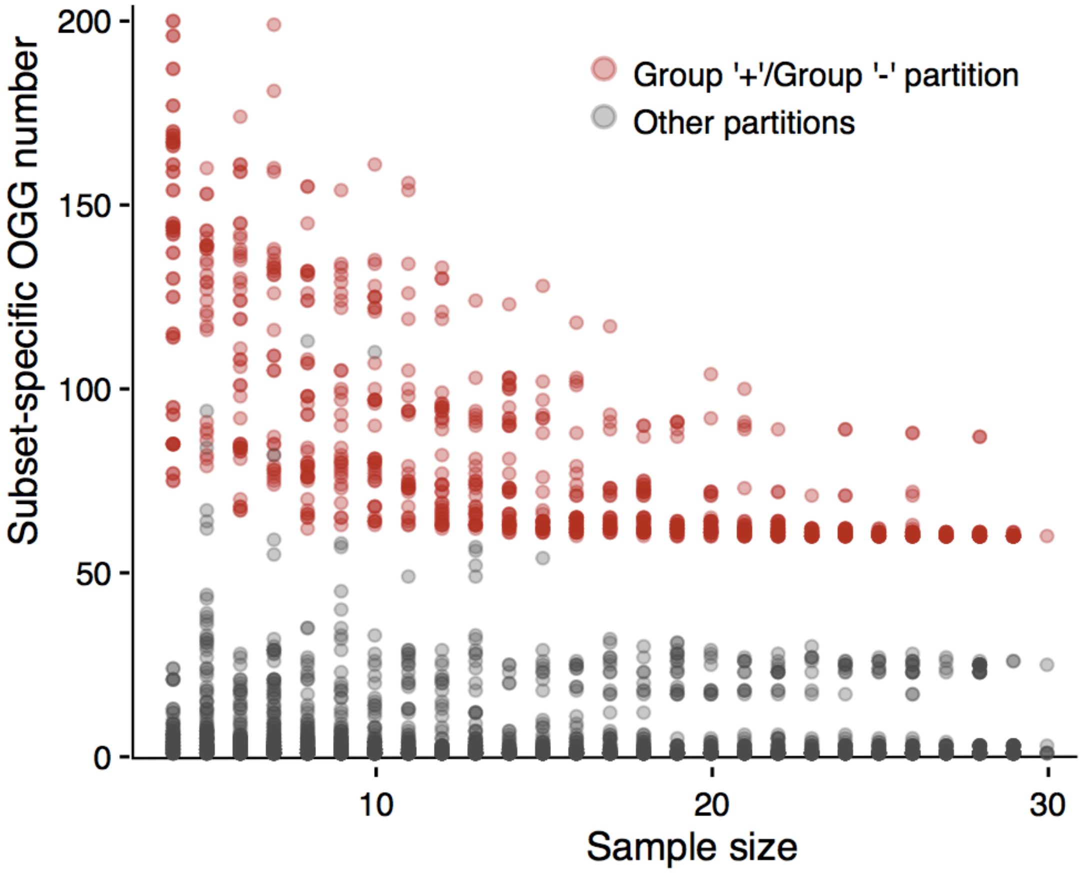

Supplementary Figure S12 | The numbers of subset-specific OGG for different *Brucella suis* partitions. The numbers for partitions consistent with the plus-group/minus-group partition are represented with pink dots, the numbers for other partitions are shown with grey dots. At small sample sizes no clear boundary between two dot clusters is seen, but it appears as the sample size increases.

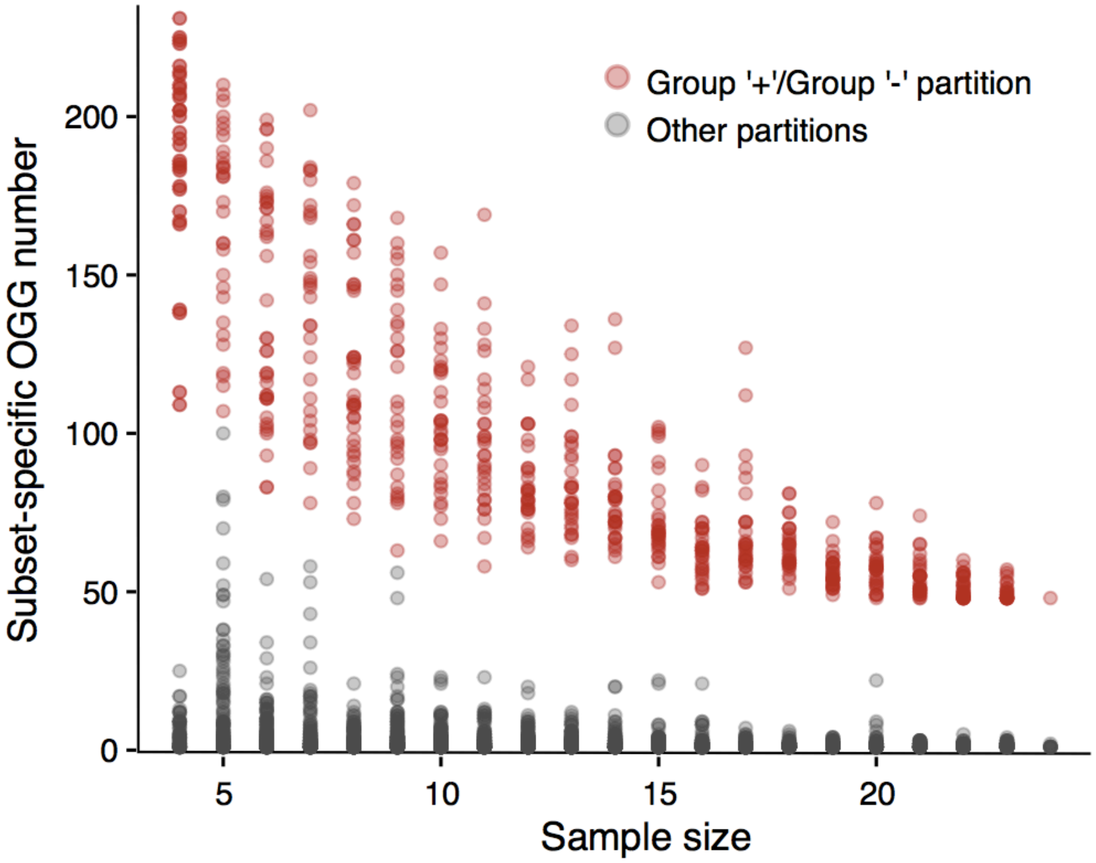

Supplementary Figure S13 | Numbers of subset-specific OGG for different *Buchnera aphidicola* partitions. The numbers for partitions consistent with the plus-group/minus-glus-Group partition are represented by pink dots, thenumbers for other partitions are shown by grey dots. As in Figure S11, the boundary between the clusters emerges as the sample size increases.

1.2 Supplementary Tables

Supplementary Table S1 | *Prochlorococcus marinus* and *Synechococcus* spp. strains used in the study.

Species	Strain	Assembly
Synechococcus sp.	KORDI-100	GCF_000737535.1
Synechococcus sp.	CC9616	GCF_000515235.1
Synechococcus sp.	KORDI-52	GCF_000737595.1
Synechococcus sp.	WH_8109	GCF_000161795.2
Synechococcus sp.	WH_8103	GCF_001182765.1
Synechococcus sp.	WH_8102	GCF_000195975.1
Synechococcus sp.	CC9605	GCF_000012625.1
Synechococcus sp.	KORDI-49	GCF_000737575.1
Prochlorococcus marinus minus-group	GP2	GCF_000759885.1
Prochlorococcus marinus minus-group	MIT_9302	GCF_000759975.1
Prochlorococcus marinus minus-group	MIT_9215	GCF_000018065.1

Prochlorococcus marinus minus-group	MIT_9314	GCF_000760035.1
Prochlorococcus marinus plus-group	MIT_1312	GCF_001632005.1
Prochlorococcus marinus minus-group	MIT_9311	GCF_000760015.1
Prochlorococcus marinus minus-group	SCGC_AAA795-I06	GCF_001180265.1
Prochlorococcus marinus minus-group	MED4	GCF_000011465.1
Prochlorococcus marinus plus-group	MIT9313	GCF_000011485.1
Prochlorococcus marinus minus-group	SS51	GCF_000760355.1
Prochlorococcus marinus minus-group	LG	GCF_000760155.1
Prochlorococcus marinus minus-group	CCMP1375_SS120	GCF_000007925.1
Prochlorococcus marinus plus-group	MIT_1313	GCF_001632065.1
Prochlorococcus marinus minus-group	MIT_9515	GCF_000015665.1
Prochlorococcus marinus minus-group	SCGC_AAA795-F05	GCF_001180245.1
Prochlorococcus marinus minus-group	AS9601	GCF_000015645.1
Prochlorococcus marinus plus-group	MIT_1323	GCF_001632025.1
Prochlorococcus marinus minus-group	MIT_9107	GCF_000759855.1
Prochlorococcus marinus minus-group	EQPAC1	GCF_000759875.1
Prochlorococcus marinus plus-group	MIT_1320	GCF_001632075.1
Prochlorococcus marinus minus-group	MIT_9321	GCF_000760055.1
Prochlorococcus marinus minus-group	MIT_9312	GCF_000012645.1
Prochlorococcus marinus minus-group	MIT_9123	GCF_000759935.1
Prochlorococcus marinus minus-group	SS35	GCF_000760275.1
Prochlorococcus marinus minus-group	SB	GCF_000760115.1
Prochlorococcus marinus minus-group	NATL1A	GCF_000015685.1
Prochlorococcus marinus minus-group	MIT_9116	GCF_000759865.1
Prochlorococcus marinus plus-group	MIT_1342	GCF_001632145.1
Prochlorococcus marinus plus-group	MIT_1327	GCF_001632125.1
Prochlorococcus marinus minus-group	MIT_9401	GCF_000760095.1
Prochlorococcus marinus minus-group	SCGC_AAA795-M23	GCF_001180325.1
Prochlorococcus marinus minus-group	MIT_9202	GCF_000158595.1
Prochlorococcus marinus minus-group	NATL2A	GCF_000012465.1
Prochlorococcus marinus minus-group	MIT_9322	GCF_000760075.1
Prochlorococcus marinus minus-group	MIT9211	GCF_000018585.1
Prochlorococcus marinus plus-group	MIT_9303	GCF_000015705.1
Prochlorococcus marinus minus-group	MIT_9201	GCF_000759955.1
Prochlorococcus marinus plus-group	HNLC2	GCF_001632045.1

Prochlorococcus marinus minus-group	SCGC_AAA795-I15	GCF_001180285.1
Prochlorococcus marinus minus-group	PAC1	GCF_000760235.1
Prochlorococcus marinus minus-group	SCGC_AAA795-J16	GCF_001180305.1
Prochlorococcus marinus minus-group	MIT_9301	GCF_000015965.1

Supplementary Table S2 | *Streptococcus equi* strains used in the study.

**Species**	**Strain**	**Assembly**
Streptococcus equi Sz group	Sz105	GCF_000876195.1
Streptococcus equi EQUI group	EQUI0203	GCF_001178445.1
Streptococcus equi EQUI group	EQUI0118	GCF_001175425.1
Streptococcus equi Sz group	Sz5	GCF_000876355.1
Streptococcus equi EQUI group	EQUI0155	GCF_001177125.1
Streptococcus equi EQUI group	EQUI0039	GCF_001175585.1
Streptococcus equi EQUI group	CF22	GCF_001509635.1
Streptococcus equi EQUI group	EQUI0038	GCF_001175305.1
Streptococcus equi EQUI group	E12	GCF_001645285.1
Streptococcus equi EQUI group	EQUI0234	GCF_001177665.1
Streptococcus equi EQUI group	EQUI0007	GCF_001178765.1
Streptococcus equi Sz group	SzAM35	GCF_000876275.1
Streptococcus equi EQUI group	EQUI0004	GCF_001179085.1
Streptococcus equi EQUI group	ATCC_33398	GCF_900156215.1
Streptococcus equi EQUI group	1-8	GCF_001510555.1
Streptococcus equi EQUI group	EQUI0197	GCF_001176465.1
Streptococcus equi Sz group	SzAM60	GCF_000876285.1
Streptococcus equi EQUI group	EQUI0088	GCF_001175285.1
Streptococcus equi EQUI group	EQUI0167	GCF_001178125.1
Streptococcus equi EQUI group	EQUI0087	GCF_001177625.1
Streptococcus equi EQUI group	EQUI0086	GCF_001177285.1
Streptococcus equi EQUI group	DSM_20561	GCF_900110405.1
Streptococcus equi EQUI group	Lex90	GCF_001645315.1
Streptococcus equi EQUI group	EQUI0002	GCF_001177525.1
Streptococcus equi Sz group	Sz12is	GCF_000876305.1
Streptococcus equi EQUI group	EQUI0111	GCF_001178185.1
Streptococcus equi EQUI group	EQUI0020	GCF_001179145.1
Streptococcus equi EQUI group	EQUI0107	GCF_001179205.1

Streptococcus equi EQUI group	EQUI0005	GCF_001179425.1
Streptococcus equi EQUI group	EQUI0223	GCF_001179445.1
Streptococcus equi EQUI group	EQUI0160	GCF_001178525.1
Streptococcus equi EQUI group	EQUI0221	GCF_001179045.1
Streptococcus equi EQUI group	EQUI0109	GCF_001177805.1
Streptococcus equi Sz group	Sz35	GCF_000876365.1
Streptococcus equi EQUI group	EQUI0224	GCF_001178545.1
Streptococcus equi EQUI group	EQUI0222	GCF_001179185.1
Streptococcus equi EQUI group	EQUI0010	GCF_001175845.1
Streptococcus equi EQUI group	EQUI0140	GCF_001179305.1
Streptococcus equi EQUI group	EQUI0100	GCF_001178785.1
Streptococcus equi EQUI group	EQUI0179	GCF_001178665.1
Streptococcus equi EQUI group	EQUI0205	GCF_001179465.1
Streptococcus equi EQUI group	EQUI0130	GCF_001176145.1
Streptococcus equi EQUI group	EQUI0235	GCF_001177885.1
Streptococcus equi EQUI group	EQUI0177	GCF_001179165.1
Streptococcus equi Sz group	Sz57	GCF_000876375.1
Streptococcus equi EQUI group	EQUI0142	GCF_001178945.1
Streptococcus equi EQUI group	EQUI0218	GCF_001178345.1
Streptococcus equi EQUI group	EQUI0162	GCF_001179285.1
Streptococcus equi EQUI group	EQUI0214	GCF_001177085.1
Streptococcus equi EQUI group	EQUI0238	GCF_001176025.1
Streptococcus equi EQUI group	EQUI0117	GCF_900004825.1
Streptococcus equi EQUI group	EQUI0240	GCF_001175405.1
Streptococcus equi EQUI group	EQUI0161	GCF_001178505.1
Streptococcus equi EQUI group	EQUI0219	GCF_001177385.1
Streptococcus equi EQUI group	HO51380626	GCF_001179245.1
Streptococcus equi EQUI group	ASM164529v1	GCF_001645295.1
Streptococcus equi EQUI group	EQUI0188	GCF_001178645.1
Streptococcus equi EQUI group	EQUI0034	GCF_001178025.1
Streptococcus equi EQUI group	EQUI0176	GCF_001179365.1
Streptococcus equi EQUI group	EQUI0029	GCF_001176285.1
Streptococcus equi EQUI group	EQUI0229	GCF_001176385.1
Streptococcus equi EQUI group	EQUI0120	GCF_001179025.1
Streptococcus equi EQUI group	EQUI0135	GCF_001177305.1

Streptococcus equi EQUI group	EQUI0182	GCF_001179265.1
Streptococcus equi EQUI group	EQUI0137	GCF_001177425.1
Streptococcus equi EQUI group	EQUI0183	GCF_001179385.1
Streptococcus equi Sz group	SzS31A1	GCF_000445225.2
Streptococcus equi EQUI group	EQUI0054	GCF_001177865.1
Streptococcus equi EQUI group	EQUI0036	GCF_001178905.1
Streptococcus equi EQUI group	EQUI0012	GCF_001176565.1
Streptococcus equi EQUI group	EQUI0046	GCF_001177245.1
Streptococcus equi EQUI group	EQUI0105	GCF_001179105.1
Streptococcus equi EQUI group	EQUI0022	GCF_001178405.1
Streptococcus equi EQUI group	EQUI0076	GCF_001179405.1
Streptococcus equi EQUI group	EQUI0231	GCF_001176925.1
Streptococcus equi EQUI group	EQUI0019	GCF_001179325.1
Streptococcus equi EQUI group	EQUI0068	GCF_001176525.1
Streptococcus equi EQUI group	F43	GCF_001645305.1
Streptococcus equi EQUI group	EQUI0074	GCF_001177485.1
Streptococcus equi EQUI group	EQUI0070	GCF_001178085.1
Streptococcus equi EQUI group	EQUI0017	GCF_001177825.1
Streptococcus equi EQUI group	EQUI0077	GCF_001178565.1
Streptococcus equi EQUI group	EQUI0082	GCF_001176325.1
Streptococcus equi EQUI group	EQUI0085	GCF_001177025.1
Streptococcus equi EQUI group	EQUI0078	GCF_001175245.1
Streptococcus equi EQUI group	EQUI0052	GCF_001177965.1
Streptococcus equi EQUI group	EQUI0121	GCF_001179225.1
Streptococcus equi EQUI group	EQUI0125	GCF_001178045.1
Streptococcus equi EQUI group	EQUI0202	GCF_001178705.1
Streptococcus equi EQUI group	EQUI0122	GCF_001178465.1
Streptococcus equi EQUI group	EQUI0049	GCF_001178925.1
Streptococcus equi EQUI group	EQUI0050	GCF_001178105.1
Streptococcus equi EQUI group	EQUI0079	GCF_001175545.1

Supplementary Table S3 | *Buchnera aphidicola* strains used in the study.

**Species**	**Strain**	**Assembly**
Buchnera aphidicola	Bp (*Baizongia pistaciae*)	GCF_000007725.1
Buchnera aphidicola	TLW03 (*Acyrthosiphon pisum*)	GCF_000183245.1

Buchnera aphidicola	JF98 (*Acyrthosiphon pisum*)	GCF_000183305.1
Buchnera aphidicola	LL01 (*Acyrthosiphon pisum*)	GCF_000183225.1
Buchnera aphidicola	Ua (*Uroleucon ambrosiae*)	GCF_000225465.1
Buchnera aphidicola	(*Diuraphis noxia*)	GCF_001700895.1
Buchnera aphidicola	BAg (*Aphis glycines*)	GCF_001280225.1
Buchnera aphidicola	JF99 (*Acyrthosiphon pisum*)	GCF_000183285.1
Buchnera aphidicola	Sg (*Schizaphis graminum*)	GCF_000007365.1
Buchnera aphidicola	LSR1 (*Acyrthosiphon pisum*)	GCF_000174075.1
Buchnera aphidicola	5A (*Acyrthosiphon pisum*)	GCF_000021085.1
Buchnera aphidicola	Tuc7 (*Acyrthosiphon pisum*)	GCF_000021065.1
Buchnera aphidicola	APS (*Acyrthosiphon pisum*)	GCF_000009605.1
Buchnera aphidicola	G006 (*Myzus persicae*)	GCF_001939165.1
Buchnera aphidicola	Ak (*Acyrthosiphon kondoi*)	GCF_000225445.1
Buchnera aphidicola	(*Cinara tujafilina*)	GCF_000217635.1
Buchnera aphidicola	G002 (*Myzus persicae*)	GCF_000521565.1
Buchnera aphidicola	F009 (*Myzus persicae*)	GCF_000521585.1
Buchnera aphidicola	BCc	GCF_000090965.1
Buchnera aphidicola	BCiconfinis (*Cinara confinis*)	GCF_900128735.1
Buchnera aphidicola	BCipseudotaxifoliae (*Cinara pseudotaxifoliae*)	GCF_900128595.1
Buchnera aphidicola	Bcifornacula	GCF_900128725.1
Buchnera aphidicola	SC (*Schlechtendalia chinensis*)	GCF_001648115.1
Buchnera aphidicola	BTs (*Tuberolachnus salignus*)	GCF_900016785.1

Supplementary Table S4 | *Yersinia pestia and Y. pseudotuberculosis* strains used in the study.

**Species**	**Strain**	**Assembly**
Yersinia pestis	PY-42	GCF_000269285.2
Yersinia pestis	2506	GCF_000323225.1
Yersinia pestis	CMCC27002	GCF_000324085.1
Yersinia pestis	MGJZ12	GCF_000325225.1
Yersinia pestis	CMCC106002	GCF_000323925.1
Yersinia pestis	1413	GCF_001188935.1
Yersinia pestis	SCPM-O-DNA-02	GCF_001972515.1
Yersinia pestis	G1670E	GCF_000382505.1
Yersinia pestis	PBM19	GCF_000834235.1
Yersinia pestis	PY-94	GCF_000269425.1
Yersinia pestis	Pestoides_B	GCF_000834925.1

Yersinia pestis	CMCC67001	GCF_000324305.1
Yersinia pestis	SCPM-O-B-6304	GCF_001294885.1
Yersinia pestis	KIM5	GCF_000970105.1
Yersinia pestis	620024	GCF_000323485.1
Yersinia pestis	Yp1945	GCF_001509865.1
Yersinia pestis	AS200901434	GCF_000255875.1
Yersinia pestis	945	GCF_000323665.1
Yersinia pestis	F1946001	GCF_000324825.1
Yersinia pestis	Yp2126	GCF_001509855.1
Yersinia pestis	7338	GCF_000323565.1
Yersinia pestis	CMCC31004	GCF_000324105.1
Yersinia pestis	PY-14	GCF_000268485.2
Yersinia pestis	D1991004	GCF_000324745.1
Yersinia pestis	PY-60	GCF_000268905.1
Yersinia pestis	PY-09	GCF_000268425.1
Yersinia pestis	SCPM-O-B-6706	GCF_000950945.1
Yersinia pestis	34008	GCF_000323305.1
Yersinia pestis	KM-816	GCF_001613895.1
Yersinia pestis	CMCC96001	GCF_000324565.1
Yersinia pestis	CMCC99103	GCF_000324605.1
Yersinia pestis	CMCC05009	GCF_000323845.1
Yersinia pestis	Pestoides_F	GCF_000016445.1
Yersinia pestis	PY-76	GCF_000269025.1
Yersinia pestis	M-978	GCF_001613885.1
Yersinia pestis	SCPM-O-B-6904	GCF_001294825.1
Yersinia pestis	1412	GCF_001188695.1
Yersinia pestis	Nepal516	GCF_000182485.1
Yersinia pestis	AS200901539	GCF_000255755.1
Yersinia pestis	Pestoides_A	GCF_000182545.1
Yersinia pestis	I1991001	GCF_000325045.1
Yersinia pestis	C1975003	GCF_000323745.1
Yersinia pestis	KIM_10v	GCF_001261975.1
Yersinia pestis	E1979001	GCF_000169695.1
Yersinia pestis	9	GCF_000323625.1
Yersinia pestis	YN663	GCF_000325485.1

Yersinia pestis	SCPM-O-B-6213	GCF_001295025.1
Yersinia pestis	PEXU2	GCF_000182525.1
Yersinia pestis	KIM	GCF_000006645.1
Yersinia pestis	CMCC92004	GCF_000324485.1
Yersinia pseudotuberculosis	Pa3606	GCF_000834945.1
Yersinia pseudotuberculosis	Y428	GCF_001123345.1
Yersinia pseudotuberculosis	IP32953	GCF_000834295.1
Yersinia pseudotuberculosis	CEB14_0017	GCF_000613105.1
Yersinia pseudotuberculosis	N912	GCF_001144105.1
Yersinia pseudotuberculosis	IP33038	GCF_001125265.1
Yersinia pseudotuberculosis	B-6864	GCF_000344015.1
Yersinia pseudotuberculosis	IP32938	GCF_001319665.1
Yersinia pseudotuberculosis	R819	GCF_001140625.1
Yersinia pseudotuberculosis	SP93422	GCF_001244855.1
Yersinia pseudotuberculosis	IP32670	GCF_001220805.1
Yersinia pseudotuberculosis	IP32544	GCF_001110725.1
Yersinia pseudotuberculosis	B-6862	GCF_000343975.1
Yersinia pseudotuberculosis	No5	GCF_001124425.1
Yersinia pseudotuberculosis	OK6088	GCF_001319885.1
Yersinia pseudotuberculosis	B-6865	GCF_000344035.1
Yersinia pseudotuberculosis	IP32921	GCF_001319685.1
Yersinia pseudotuberculosis	YPIII	GCF_000834375.1
Yersinia pseudotuberculosis	ATCC_4284	GCF_000754965.1
Yersinia pseudotuberculosis	PB1/+	GCF_000834475.1
Yersinia pseudotuberculosis	1231	GCF_001108005.1
Yersinia pseudotuberculosis	NBRC_105692	GCF_000511675.1
Yersinia pseudotuberculosis	PT682	GCF_001319725.1
Yersinia pseudotuberculosis	IH111554	GCF_001104045.1
Yersinia pseudotuberculosis	PB1/+	GCF_000020085.1
Yersinia pseudotuberculosis	1	GCF_000834435.1
Yersinia pseudotuberculosis	B-7194	GCF_000403705.1
Yersinia pseudotuberculosis	YP4713	GCF_900092345.1
Yersinia pseudotuberculosis	IP33177	GCF_001136125.1
Yersinia pseudotuberculosis	OK5586	GCF_001166025.1
Yersinia pseudotuberculosis	B-6866	GCF_000344055.1

Yersinia pseudotuberculosis	IP33054	GCF_001157205.1
Yersinia pseudotuberculosis	IP32463	GCF_001172325.1
Yersinia pseudotuberculosis	MD67	GCF_000834355.1
Yersinia pseudotuberculosis	Y722	GCF_001155645.1
Yersinia pseudotuberculosis	260	GCF_001319765.1
Yersinia pseudotuberculosis	IP33250	GCF_001319785.1
Yersinia pseudotuberculosis	IP_32953	GCF_000047365.1
Yersinia pseudotuberculosis	2888	GCF_001150985.1
Yersinia pseudotuberculosis	IP32881	GCF_001319705.1
Yersinia pseudotuberculosis	B-6796	GCF_000343955.1
Yersinia pseudotuberculosis	B-6863	GCF_000343995.1
Yersinia pseudotuberculosis	MW109-2	GCF_001136465.1
Yersinia pseudotuberculosis	EP2/+	GCF_000834415.1
Yersinia pseudotuberculosis	YPIII	GCF_000019465.1
Yersinia pseudotuberculosis	Y716	GCF_001244655.1
Yersinia pseudotuberculosis	IP_31758	GCF_000016945.1

Supplementary Table S5 | *Brucella suis* strains used in the study.

**Species**	**Strain**	**Assembly**
Brucella suis	bv. 1 str. S2	GCF_000209635.2
Brucella suis	bv. 3 str. 686	GCF_000740255.1
Brucella suis	bv. 2 str. BCB032	GCF_000292105.1
Brucella suis	bv. 1 str. BCB025	GCF_000292005.1
Brucella suis	F5/05-1	GCF_000371185.1
Brucella suis	04-0115	GCF_000480055.1
Brucella suis	06-791-1309	GCF_000480035.1
Brucella suis	F12/02	GCF_000371165.1
Brucella suis	513UK	GCF_000740235.1
Brucella suis	92/63	GCF_000366225.1
Brucella suis	F4/06-146	GCF_000365705.1
Brucella suis	bv. 2 str. Bs364CITA	GCF_000698325.1
Brucella suis	Thompsen	GCF_000742055.1
Brucella suis	bv. 2 str. PT09172	GCF_000698285.1
Brucella suis	F8/06-3	GCF_000371305.1
Brucella suis	2004000577	GCF_001715425.1

Brucella suis	BSQ	GCF_000741995.1
Brucella suis	F5/05-4	GCF_000371205.1
Brucella suis	19	GCF_001594165.1
Brucella suis	F7/06-2	GCF_000371245.1
Brucella suis	bv. 2 str. Bs396CITA	GCF_000698345.1
Brucella suis	06-997-1672	GCF_000480135.1
Brucella suis	BSP	GCF_000740435.1
Brucella suis	Human/AR/US/1981	GCF_000875695.1
Brucella suis	CNGB 247	GCF_000371145.1
Brucella suis	bv. 1 str. S2	GCF_000600055.1
Brucella suis	1330	GCF_000742005.1
Brucella suis	VBI22	GCF_000236255.1
Brucella suis	bv. 3 str. 686	GCF_000157775.1
Brucella suis	bv. 5 str. 513	GCF_000157755.1

Supplementary Table S6 | *Burkholderia mallei* and *B. pseudomallei* strains used in the study.

**Species**	**Strain**	**Assembly**
Burkholderia mallei	Kweiyang#4	GCF_001608335.1
Burkholderia mallei	FMH_23344	GCF_000755785.1
Burkholderia mallei	BMQ	GCF_000755885.1
Burkholderia mallei	Turkey4	GCF_001278925.1
Burkholderia mallei	China5	GCF_000757315.2
Burkholderia mallei	Ivan	GCF_000986905.1
Burkholderia mallei	SR092700I	GCF_000756865.1
Burkholderia mallei	2000031281	GCF_000565425.1
Burkholderia mallei	Turkey5	GCF_001279195.1
Burkholderia mallei	2002721276	GCF_000959625.1
Burkholderia mallei	Turkey2	GCF_001279325.1
Burkholderia mallei	strain_6	GCF_000565385.1
Burkholderia mallei	Bahrain1	GCF_001729545.1
Burkholderia mallei	Turkey7	GCF_001279185.1
Burkholderia mallei	Turkey10	GCF_001279265.1
Burkholderia mallei	GB8_horse_4	GCF_000167635.1
Burkholderia mallei	NCTC_10229	GCF_000015605.1
Burkholderia mallei	ATCC_23344	GCF_000011705.1

Burkholderia mallei	6	GCF_000755845.1
Burkholderia mallei	BURK081	GCF_001608415.1
Burkholderia mallei	Budapest	GCF_001608345.1
Burkholderia mallei	2002734299	GCF_000959165.1
Burkholderia mallei	PRL-20	GCF_000169875.1
Burkholderia mallei	23344	GCF_000755865.1
Burkholderia mallei	NCTC_10247	GCF_000015625.1
Burkholderia mallei	Turkey1	GCF_001279275.1
Burkholderia mallei	3712	GCF_001715185.1
Burkholderia mallei	A193	GCF_000565465.1
Burkholderia mallei	102	GCF_001608365.1
Burkholderia mallei	India86-567-2	GCF_000959465.1
Burkholderia mallei	SAVP1	GCF_000015465.1
Burkholderia mallei	A188	GCF_000565445.2
Burkholderia mallei	BURK080	GCF_001608455.1
Burkholderia mallei	Turkey3	GCF_001279245.1
Burkholderia mallei	Turkey8	GCF_001279165.1
Burkholderia mallei	strain_11	GCF_000565405.2
Burkholderia mallei	3076	GCF_001831355.1
Burkholderia mallei	NCTC_10247	GCF_000762285.1
Burkholderia mallei	Turkey9	GCF_001279255.1
Burkholderia mallei	China_7	GCF_000565365.2
Burkholderia mallei	FMH	GCF_000152385.1
Burkholderia mallei	2002721280	GCF_000153085.1
Burkholderia mallei	11	GCF_000959405.1
Burkholderia mallei	2000031063	GCF_000756025.1
Burkholderia pseudomallei	MSHR346	GCF_000756105.1
Burkholderia pseudomallei	TSV28	GCF_000775085.1
Burkholderia pseudomallei	NAU14A-1	GCF_001978405.1
Burkholderia pseudomallei	MSHR3042	GCF_001979335.1
Burkholderia pseudomallei	MSHR4504	GCF_001980315.1
Burkholderia pseudomallei	MSHR1688	GCF_001979755.1
Burkholderia pseudomallei	BGH	GCF_000757165.1
Burkholderia pseudomallei	C36	GCF_001192415.1
Burkholderia pseudomallei	4226b	GCF_001981185.1

Burkholderia pseudomallei	vgh16R	GCF_001277875.1
Burkholderia pseudomallei	D10_7310-3154	GCF_001327175.1
Burkholderia pseudomallei	708a	GCF_001327395.1
Burkholderia pseudomallei	BDD	GCF_000756905.1
Burkholderia pseudomallei	612	GCF_001320395.1
Burkholderia pseudomallei	MSHR4032	GCF_000774335.1
Burkholderia pseudomallei	RNS3Bp1	GCF_001446125.1
Burkholderia pseudomallei	E8	GCF_001327435.1
Burkholderia pseudomallei	PHLS9	GCF_001446095.1
Burkholderia pseudomallei	Ubon-P19-Bp11	GCF_001978015.1
Burkholderia pseudomallei	NAU21B-13	GCF_001637015.1
Burkholderia pseudomallei	10./96	GCF_001195465.1
Burkholderia pseudomallei	OS	GCF_000401095.1
Burkholderia pseudomallei	2381a	GCF_001981045.1
Burkholderia pseudomallei	MSHR3974	GCF_001980265.1
Burkholderia pseudomallei	MSHR3841	GCF_001980175.1
Burkholderia pseudomallei	2002721787	GCF_001976245.1
Burkholderia pseudomallei	CMS	GCF_000402055.1
Burkholderia pseudomallei	Songkhla34W2	GCF_001446175.1
Burkholderia pseudomallei	Ubon-P19-Bp45	GCF_001977225.1
Burkholderia pseudomallei	PB08298010	GCF_000959345.1
Burkholderia pseudomallei	MSHR1218	GCF_001979215.1
Burkholderia pseudomallei	HA	GCF_001182305.1
Burkholderia pseudomallei	C55	GCF_001321625.1
Burkholderia pseudomallei	TSV_43	GCF_000774255.1
Burkholderia pseudomallei	112	GCF_000170555.1
Burkholderia pseudomallei	MSHR3709	GCF_000775025.1
Burkholderia pseudomallei	4095a	GCF_001981165.1
Burkholderia pseudomallei	MSHR3763	GCF_001975145.1
Burkholderia pseudomallei	MSHR4750	GCF_001980485.1
Burkholderia pseudomallei	MSHR62	GCF_000770395.1
Burkholderia pseudomallei	EY9	GCF_001327455.1
Burkholderia pseudomallei	RF85Bp37	GCF_001446115.1
Burkholderia pseudomallei	PHLS_112	GCF_000757015.2
Burkholderia pseudomallei	MSHR0465A	GCF_001978185.1

Burkholderia pseudomallei	C6	GCF_001320905.1
Burkholderia pseudomallei	NAU44A-6	GCF_001979045.1
Burkholderia pseudomallei	NAU21B-7	GCF_001978785.1
Burkholderia pseudomallei	14	GCF_000170435.1
Burkholderia pseudomallei	NAU33A-6	GCF_001978905.1
Burkholderia pseudomallei	BDT	GCF_000757105.1

Supplementary Table S7 | *Escherichia coli* and *Shigella* spp. strains used in the study.

**Species**	**Strain**	**Assembly**
Escherichia coli	swine4	GCF_001614875.1
Escherichia coli	MNCRE22	GCF_001443095.1
Escherichia coli	685	GCF_001894705.1
Escherichia coli	466	GCF_001892105.1
Escherichia coli	117	GCF_001419845.1
Escherichia coli	CFSAN033951	GCF_001696245.1
Escherichia coli	K6590	GCF_000619385.1
Escherichia coli	402097_aEPEC	GCF_001284245.1
Escherichia coli	2011C-3198	GCF_001645225.1
Escherichia coli	PPECC42	GCF_001542545.1
Escherichia coli	AG100_Sample3	GCF_900096795.1
Escherichia coli	ED657	GCF_900014325.1
Escherichia coli	1351	GCF_001647405.1
Escherichia coli	F1-8-ERB4	GCF_900092915.1
Escherichia coli	EC1	GCF_001704835.1
Escherichia coli	C227-11	GCF_000986765.1
Escherichia coli	AY64	GCF_001484265.1
Escherichia coli	VL2395	GCF_001749625.1
Escherichia coli	EC1	GCF_000708145.1
Escherichia coli	78B5	GCF_001942085.1
Escherichia coli	STEC_2499	GCF_001606915.1
Escherichia coli	TOP2386	GCF_000397505.1
Escherichia coli	swine6	GCF_001614965.1
Escherichia coli	AG100_Sample1	GCF_900096825.1
Escherichia coli	Escherichia_coli_O26:H11_21765	GCF_900000205.1
Escherichia coli	85H4	GCF_001942065.1

Escherichia coli	scaffolds_20160020	GCF_900093915.1
Escherichia coli	H5	GCF_001900415.1
Escherichia coli	566	GCF_001894545.1
Escherichia coli	PA4	GCF_000303875.2
Escherichia coli	CFSAN025125	GCF_001572565.1
Escherichia coli	660	GCF_001893555.1
Escherichia coli	FHI100	GCF_000951955.1
Escherichia coli	DH1Ec095	GCF_001183645.1
Escherichia coli	PARC444	GCF_001309595.1
Escherichia coli	ED766	GCF_900015875.1
Escherichia coli	NE037	GCF_000303835.2
Escherichia coli	HS115	GCF_001561895.1
Escherichia coli	REL606	GCF_000017985.1
Escherichia coli	KTE75	GCF_000351685.1
Escherichia coli	STEC_2620	GCF_001607635.1
Escherichia coli	4-203-08_S3_C1	GCF_000714025.1
Escherichia coli	ATCC_BAA-2215	GCF_000506845.1
Escherichia coli	STEC_757	GCF_001608085.1
Escherichia coli	507	GCF_001892745.1
Escherichia coli	CFSAN026820	GCF_001191435.1
Escherichia coli	E1140	GCF_000622285.1
Escherichia coli	NG-7574_Bell_ERRESI	GCF_900041715.1
Escherichia coli	554	GCF_001892595.1
Escherichia coli	STEC_196	GCF_001607415.1
Escherichia coli	449	GCF_001891315.1
Escherichia coli	SF-501	GCF_001881045.1
Escherichia coli	BIDMC_37	GCF_000492275.1
Escherichia coli	1409160003	GCF_001692795.1
Escherichia coli	sheep16	GCF_001616455.1
Escherichia coli	100197	GCF_001912475.1
Escherichia coli	EC1636	GCF_001650605.1
Escherichia coli	G303	GCF_001576095.1
Escherichia coli	WCE307	GCF_001640245.1
Escherichia coli	FORC_041	GCF_001886935.1
Escherichia coli	C3026	GCF_001559675.1

Escherichia coli	CR694	GCF_000814525.1
Escherichia coli	ED142	GCF_900015835.1
Escherichia coli	H1	GCF_001900775.1
Escherichia coli	655	GCF_001892865.1
Escherichia coli	628	GCF_001892135.1
Escherichia coli	upec-80	GCF_000776035.1
Escherichia coli	cloneA_i1	GCF_000233675.1
Escherichia coli	scaffolds_20160018	GCF_900093975.1
Escherichia coli	STEC_1363	GCF_001607155.1
Escherichia coli	601028	GCF_001912525.1
Escherichia coli	STEC_1299	GCF_001606785.1
Escherichia coli	713	GCF_001894125.1
Escherichia coli	GN02140	GCF_001519775.1
Escherichia coli	OLC-455	GCF_000948715.1
Escherichia coli	457	GCF_001891545.1
Escherichia coli	RiKo_2331/09	GCF_001637745.1
Escherichia coli	210205630	GCF_001679985.1
Escherichia coli	STEC_2074	GCF_001606955.1
Escherichia coli	CD26	GCF_001571685.1
Escherichia coli	503891_aEPEC	GCF_001286285.1
Escherichia coli	500	GCF_001894025.1
Escherichia coli	H2	GCF_001900795.1
Escherichia coli	4626_wz	GCF_001931785.1
Escherichia coli	ICBEcBL-II-13	GCF_001982585.1
Escherichia coli	C1	GCF_001900295.1
Escherichia coli	No.12	GCF_001865915.1
Escherichia coli	1.ECAF83.1	GCF_001269305.1
Escherichia coli	549	GCF_001893915.1
Escherichia coli	350	GCF_001890265.1
Escherichia coli	upec-284	GCF_000777515.1
Escherichia coli	cattle18	GCF_001614775.1
Escherichia coli	SRCC_1675	GCF_001612495.1
Escherichia coli	GN02148	GCF_001519755.1
Escherichia coli	711	GCF_001893775.1
Escherichia coli	tEC	GCF_001596855.1

Escherichia coli	IMT31351	GCF_001282155.1
Escherichia coli	TA143	GCF_000176615.2
Escherichia coli	AG100_Sample2	GCF_900096855.1
Escherichia coli	95.0943	GCF_000316885.2
Shigella sonnei	sh1451	GCF_900160365.1
Shigella sonnei	2090STDY5488123	GCF_900158755.1
Shigella sonnei	2090STDY5488133	GCF_900158855.1
Shigella sonnei	sh1411	GCF_900159975.1
Shigella sonnei	3626STDY6095482	GCF_900159015.1
Shigella sonnei	2090STDY5488119	GCF_900158715.1
Shigella sonnei	201400003_1	GCF_001256875.1
Shigella sonnei	20051272_1361367	GCF_001246015.1
Shigella sonnei	FDAARGOS_71	GCF_000783795.1
Shigella sonnei	sh1463	GCF_900160475.1
Shigella flexneri	2002021	GCF_000566165.1
Shigella flexneri	Shi06HN023	GCF_000565625.1
Shigella flexneri	Shi06HN344	GCF_000566465.1
Shigella flexneri	2002069	GCF_000566205.1
Shigella flexneri	2003035	GCF_000566305.1
Shigella flexneri	228	GCF_001578125.1
Shigella flexneri	Shi06HN250	GCF_000565725.1
Shigella flexneri	4S_BJ10610	GCF_000710235.1
Shigella flexneri	Shi06AH135	GCF_000565665.1
Shigella flexneri	FDAARGOS_74	GCF_000783735.1
Shigella boydii	08-2671	GCF_000815725.1
Shigella boydii	08-2671	GCF_001906365.1
Shigella boydii	Sb227	GCF_000012025.1
Shigella boydii	260_SBOY	GCF_001063095.1
Shigella boydii	102265	GCF_001906435.1
Shigella boydii	603210	GCF_001906355.1
Shigella boydii	600080	GCF_001905975.1
Shigella boydii	600266	GCF_001906105.1
Shigella boydii	1146_SBOY	GCF_001062045.1
Shigella boydii	603122	GCF_001906295.1
Shigella dysenteriae	S6554	GCF_000815515.1

Shigella dysenteriae	WRSd3	GCF_000499065.1
Shigella dysenteriae	BS1047	GCF_001017215.1
Shigella dysenteriae	CDC_74-1112	GCF_000193895.1
Shigella dysenteriae	WRSd5	GCF_000499085.1
Shigella dysenteriae	Sd197	GCF_000012005.1
Shigella dysenteriae	BCW_4871	GCF_001933095.2
Shigella dysenteriae	BCW_4872	GCF_001932995.2
Shigella dysenteriae	SD1D	GCF_000467245.1
Shigella dysenteriae	BCW_4870	GCF_001932975.2

Supplementary Table S8 | Outgroups used to construct phylogenetic trees.

**Species**	**Outgroup species**	**Assembly ID**
Prochlorococcus marinus and *Synechococcus* spp.	*Acaryochloris marina* str. MBIC11017	GCA_000018105.1
Brucella suis	*Brucella ovis* str. ATCC 25840	GCF_000016845.1
Buchnera aphidicola	*Sodalis glossinidius* str. 'morsitans'	GCF_000010085.1
Streptococcus equi	*Streptococcus gallolyticus* str. UCN34	GCF_000027185.1
Escherichia coli and *Schigella* spp.	*Salmonella enterica* subsp. enterica serovar Kentucky str. CVM29188	GCF_000170195.2
Yersinia pestis/pseudotuberculosis	*Yersinia frederiksenii* str. ATCC 33641	GCF_000168015.1
Burkholderia mallei/pseudomallei	*Burkholderia oklahomensis* str. C6786	GCF_000170375.1

Supplementary table S9 | Functions of OGGs specific for the identified groups of strains and the sizes of group-specific functional categories.

***Prochlorococcus marinus* plus-group**		***Prochlorococcus marinus* minus-group**	
Other Putative Function	260	Other Putative Function	17
Photosynthesis	7	Metabolism	3
Bioenergetics	9	Protein Binding	2
Metabolism	47		
Transport	35		
DNA binding, Translation, Replication, DNA Repair	19		
Protein Binding	8		
Streptococcus equi* subsp. *zooepidemicus		***Streptococcus equi* subsp. *equi***	
Other Putative Function	28	Other Putative Function	51
Transport	8	Transport	1
Transcription, Translation, Replication, DNA Repair	9	DNA methylation, recombination, Transcription	8

Metabolism and Bioenergetics	11	Pathogenesis	3
Protein Binding	5	Endopeptidase	1
Bacterial Immunity	5		
***1.2.1.1.1 Brucella suis* minus-group**		***Brucella suis* plus-group**	
Other Putative Function	11	Other Putative Function	20
Transcription, Translation, Replication, DNA Repair	1	Transport	4
Metabolism and Bioenergetics	4	Transcription, Translation, Replication, DNA Repair	3
		Metabolism and Bioenergetics	15
		Protein Binding	1
***1.2.1.1.2 Buchnera aphidicola* minus-group**		***1.2.1.1.3 Buchnera aphidicola* plus-group**	
Other Putative Function	1	Other Putative Function	6
Metabolism and Bioenergetics	2	Transport	3
		Transcription, Translation, Replication, DNA Repair	2
		Metabolism and Bioenergetics	31
		Protein Binding	2

Supplementary Table S10 | *Brucella suis*, GO annotations specific for the minus-group-specific OGGs.

GO term	GO definition
GO:0004612	phosphoenolpyruvate carboxykinase (ATP) activity
GO:0004351	glutamate decarboxylase activity
GO:0010181	FMN binding
GO:0016810	hydrolase activity, acting on carbon-nitrogen (but not peptide) bonds
GO:0006355	regulation of transcription, DNA-templated

Supplementary Table S11 | *Brucella suis*, GO annotations specific for the plus-group-specific OGGs.

GO term	GO definition
GO:0005215	transporter activity
GO:0016787	hydrolase activity
GO:0006865	amino acid transport
GO:0006813	potassium ion transport
GO:0008908	isochorismatase activity
GO:0016757	transferase activity, transferring glycosyl groups
GO:0019310	inositol catabolic process
GO:0003824	catalytic activity
GO:0008914	leucyltransferase activity
GO:0006281	DNA repair

GO:0006629	lipid metabolic process
GO:0000155	phosphorelay sensor kinase activity
GO:0016772	transferase activity, transferring phosphorus-containing groups
GO:0003887	DNA-directed DNA polymerase activity
GO:0016491	oxidoreductase activity
GO:0005975	carbohydrate metabolic process
GO:0016021	integral component of membrane
GO:0008152	metabolic process
GO:0006364	rRNA processing
GO:0004854	xanthine dehydrogenase activity
GO:0030976	thiamine pyrophosphate binding
GO:0016810	hydrolase activity, acting on carbon-nitrogen (but not peptide) bonds
GO:0016829	lyase activity
GO:0008976	polyphosphate kinase activity
GO:0008484	sulfuric ester hydrolase activity
GO:0015614	D-xylose-importing ATPase activity
GO:0016746	transferase activity, transferring acyl groups

Supplementary Table S12 | *Buchnera aphidicola*, GO annotations specific for the minus-group-specific OGGs.

GO term	GO definition
GO:0016407	acetyltransferase activity
GO:0008233	peptidase activity

Supplementary Table S13 | *Buchnera aphidicola*, GO annotations specific for the plus-group-specific OGGs.

GO term	GO definition
GO:0045261	proton-transporting ATP synthase complex, catalytic core F(1)
GO:0004146	dihydrofolate reductase activity
GO:0009263	deoxyribonucleotide biosynthetic process
GO:0006741	NADP biosynthetic process
GO:0009117	nucleotide metabolic process
GO:0004252	serine-type endopeptidase activity
GO:0019243	methylglyoxal catabolic process to D-lactate via S-lactoyl-glutathione
GO:0009116	nucleoside metabolic process
GO:0004590	orotidine-5'-phosphate decarboxylase activity

GO:0009231	riboflavin biosynthetic process
GO:0016780	phosphotransferase activity, for other substituted phosphate groups
GO:0004015	adenosylmethionine-8-amino-7-oxononanoate transaminase activity
GO:0042626	ATPase activity, coupled to transmembrane movement of substances
GO:0016787	hydrolase activity
GO:0030694	bacterial-type flagellum basal body, rod
GO:0003677	DNA binding
GO:0005355	glucose transmembrane transporter activity
GO:0004385	guanylate kinase activity
GO:0004799	thymidylate synthase activity
GO:0008703	5-amino-6-(5-phosphoribosylamino)uracil reductase activity
GO:0002949	tRNA threonylcarbamoyladenosine modification
GO:0008881	glutamate racemase activity
GO:0004019	adenylosuccinate synthase activity
GO:0052547	regulation of peptidase activity
GO:0008926	mannitol-1-phosphate 5-dehydrogenase activity
GO:0008686	3,4-dihydroxy-2-butanone-4-phosphate synthase activity
GO:0006817	phosphate ion transport
GO:0050380	undecaprenyl-diphosphatase activity
GO:0008764	UDP-N-acetylmuramoylalanine-D-glutamate ligase activity
GO:0033177	proton-transporting two-sector ATPase complex, proton-transporting domain
GO:0009401	phosphoenolpyruvate-dependent sugar phosphotransferase system
GO:0006221	pyrimidine nucleotide biosynthetic process
GO:0016765	transferase activity, transferring alkyl or aryl (other than methyl) groups
GO:0016021	integral component of membrane
GO:0055085	transmembrane transport
GO:0004363	glutathione synthase activity
GO:0003977	UDP-N-acetylglucosamine diphosphorylase activity
GO:0009349	riboflavin synthase complex
GO:0008745	N-acetylmuramoyl-L-alanine amidase activity
GO:0045263	proton-transporting ATP synthase complex, coupling factor F(o)
GO:0046933	proton-transporting ATP synthase activity, rotational mechanism
GO:0015986	ATP synthesis coupled proton transport
GO:0045261	proton-transporting ATP synthase complex, catalytic core F(1)

Supplementary Table S14 | *Streptococcus equi*, GO annotations specific for the Sz-group-specific OGGs.

GO term	GO definition
GO:0003677|GO:0006356	DNA binding|regulation of transcription, DNA-templated
GO:0043565	sequence-specific DNA binding
GO:0005515	protein binding
GO:0016787	hydrolase activity
GO:0009401	phosphoenolpyruvate-dependent sugar phosphotransferase system
GO:0008982	protein-N(PI)-phosphohistidine-sugar phosphotransferase activity
GO:0033920	6-phospho-beta-galactosidase activity
GO:0005215	transporter activity
GO:0006014	D-ribose metabolic process
GO:0044341	sodium-dependent phosphate transport
GO:0016020	membrane
GO:0015658	branched-chain amino acid transmembrane transporter activity
GO:0005524	ATP binding
GO:0043571	maintenance of CRISPR repeat elements
GO:0006281	DNA repair
GO:0043571	maintenance of CRISPR repeat elements
GO:0004252	serine-type endopeptidase activity
GO:0003951	NAD+ kinase activity
GO:0042742	defense response to bacterium
GO:0017013	protein flavinylation
GO:0055085	transmembrane transport
GO:0055085	transmembrane transport
GO:0005524	ATP binding
GO:0016301	kinase activity
GO:0005975	carbohydrate metabolic process
GO:0055085	transmembrane transport
GO:0009401	phosphoenolpyruvate-dependent sugar phosphotransferase system
GO:0016740	transferase activity
GO:0003677	DNA binding
GO:0016021	integral component of membrane
GO:0005524	ATP binding
GO:0043571	maintenance of CRISPR repeat elements
GO:0043211	carbohydrate-transporting ATPase activity
GO:0043565	sequence-specific DNA binding

GO:0004252	serine-type endopeptidase activity
GO:0016887	ATPase activity
GO:0006139	nucleobase-containing compound metabolic process
GO:0005215	transporter activity
GO:0005996	monosaccharide metabolic process
GO:0006355	regulation of transcription, DNA-templated
GO:0010468	regulation of gene expression
GO:0006355	regulation of transcription, DNA-templated
GO:0043565	sequence-specific DNA binding
GO:0016491	oxidoreductase activity

Supplementary Table S15 | *Streptococcus equi*, GO annotations specific for the EQUI -group-specific OGGs.

GO term	GO definition
GO:0003676|GO:0008168	nucleic acid binding|methyltransferase activity
GO:0016887	ATPase activity
GO:0005576|GO:0009405	extracellular region|pathogenesis
GO:0003796	lysozyme activity
GO:0004415	hyalurononglucosaminidase activity
GO:0006306	DNA methylation
GO:0006304|GO:0008168	DNA modification|methyltransferase activity
GO:0009036	Type II site-specific deoxyribonuclease activity
GO:0006310	DNA recombination
GO:0043565	sequence-specific DNA binding
GO:0003729	mRNA binding
GO:0004519	endonuclease activity
GO:0004252	serine-type endopeptidase activity

Supplementary Table S16 | *Prochlorococcus marinus*, GO annotations specific for the plus-group-specific OGGs.

GO term	GO definition
GO:0006810	transport
GO:0009523	photosystem II
GO:0055114	oxidation-reduction process
GO:0004518	nuclease activity
GO:0055114	oxidation-reduction process

GO:0055114	oxidation-reduction process
GO:0016614	oxidoreductase activity, acting on CH-OH group of donors
GO:0016020	membrane
GO:0055114	oxidation-reduction process
GO:0052699	ergothioneine biosynthetic process
GO:0005215	transporter activity
GO:0004559	alpha-mannosidase activity
GO:0003677	DNA binding
GO:0006355	regulation of transcription, DNA-templated
GO:0005215	transporter activity
GO:0016887	ATPase activity
GO:0005515	protein binding
GO:0016987	sigma factor activity
GO:0016787	hydrolase activity
GO:0055085	transmembrane transport
GO:0016491	oxidoreductase activity
GO:0051536	iron-sulfur cluster binding
GO:0006051	N-acetylmannosamine metabolic process
GO:0003700	transcription factor activity, sequence-specific DNA binding
GO:0016491	oxidoreductase activity
GO:0008745	N-acetylmuramoyl-L-alanine amidase activity
GO:0003700	transcription factor activity, sequence-specific DNA binding
GO:0015221	lipopolysaccharide transmembrane transporter activity
GO:0001522	pseudouridine synthesis
GO:0010308	acireductone dioxygenase (Ni2+-requiring) activity
GO:0009982	pseudouridine synthase activity
GO:0004252	serine-type endopeptidase activity
GO:0005524	ATP binding
GO:0005515	protein binding
GO:0005975	carbohydrate metabolic process
GO:0006810	transport
GO:0006281	DNA repair
GO:0006355	regulation of transcription, DNA-templated
GO:0006310	DNA recombination
GO:0003677|GO:0005524|GO:0016787	DNA binding|ATP binding|hydrolase activity

GO:0009523|GO:0020037	photosystem II | heme binding
GO:0005215	transporter activity
GO:0004386	helicase activity
GO:0004348	glucosylceramidase activity
GO:0009539	photosystem II reaction center
GO:0016787	hydrolase activity
GO:0009058	biosynthetic process
GO:0004252	serine-type endopeptidase activity
GO:0005515	protein binding
GO:0009055	electron carrier activity
GO:0009654	photosystem II oxygen evolving complex
GO:0016021	integral component of membrane
GO:0003824	catalytic activity
GO:0030170	pyridoxal phosphate binding
GO:0005215	transporter activity
GO:0006810	transport
GO:0016021	integral component of membrane
GO:0006810	transport
GO:0004342	glucosamine-6-phosphate deaminase activity
GO:0008168	methyltransferase activity
GO:0009022	tRNA nucleotidyltransferase activity
GO:0016740	transferase activity
GO:0006810	transport
GO:0008168	methyltransferase activity
GO:0008199	ferric iron binding
GO:0008976	polyphosphate kinase activity
GO:0000155	phosphorelay sensor kinase activity
GO:0004518	nuclease activity
GO:0005509	calcium ion binding
GO:0055085	transmembrane transport
GO:0005515	protein binding
GO:0006950	response to stress
GO:0006355	regulation of transcription, DNA-templated
GO:0047746	chlorophyllase activity
GO:0000270	peptidoglycan metabolic process

GO:0015299	solute:proton antiporter activity
GO:0006355	regulation of transcription, DNA-templated
GO:0003677	DNA binding
GO:0051082	unfolded protein binding
GO:0071949	FAD binding
GO:0016747	transferase activity, transferring acyl groups other than amino-acyl groups
GO:0005215	transporter activity
GO:0051536	iron-sulfur cluster binding
GO:0055085	transmembrane transport
GO:0090529	cell septum assembly
GO:0008168	methyltransferase activity
GO:0017174	glycine N-methyltransferase activity
GO:0006810	transport
GO:0005215	transporter activity
GO:0001522	pseudouridine synthesis
GO:0004809	tRNA (guanine-N2-)-methyltransferase activity
GO:0019509	L-methionine salvage from methylthioadenosine
GO:0043874	acireductone synthase activity
GO:0016021	integral component of membrane
GO:0030288	outer membrane-bounded periplasmic space
GO:0005216	ion channel activity
GO:0003700	transcription factor activity, sequence-specific DNA binding
GO:0006744	ubiquinone biosynthetic process
GO:0008484	sulfuric ester hydrolase activity
GO:0009512	cytochrome b6f complex
GO:0015979	photosynthesis
GO:0016832	aldehyde-lyase activity
GO:0016020	membrane
GO:0045454	cell redox homeostasis
GO:0030976	thiamine pyrophosphate binding
GO:0004352	glutamate dehydrogenase (NAD+) activity
GO:0016021	integral component of membrane
GO:0009523	photosystem II
GO:0006821	chloride transport
GO:0016021	integral component of membrane

GO:0008654	phospholipid biosynthetic process
GO:0015299	solute:proton antiporter activity
GO:0004733	pyridoxamine-phosphate oxidase activity
GO:0055085	transmembrane transport
GO:0016491	oxidoreductase activity
GO:0005215	transporter activity
GO:0030170	pyridoxal phosphate binding
GO:0016756	glutathione gamma-glutamylcysteinyltransferase activity
GO:0015031	protein transport
GO:0009523	photosystem II
GO:0004351	glutamate decarboxylase activity
GO:0008080	N-acetyltransferase activity

Supplementary Table S1z7 | *Prochlorococcus marinus*, GO annotations specific for the us-group-specific OGGs.

GO term	GO definition
GO:0016021	integral component of membrane
GO:0070694	deoxyribonucleoside 5'-monophosphate N-glycosidase activity
GO:0009116	nucleoside metabolic process
GO:0005515	protein binding
GO:0006457	protein folding

Supplementary Table S18 | $G(k)$ functions of species during the initial search for nonhomogenous groups of strains. The green cell indicates a species (*Prochlorococcus marinus*) that satisfies the strong criterion. The yellow cells mark species satisfying the weak criterion.

Species	1	2	3	4	5	6	7	8	9	10	11	12	13	14	15	16
Acinetobacter baumannii	1403	433	310	167	133	76	167	61	51	30	32	40	51	102	312	2443
Actinobacillus pleuropneumoniae	287	144	75	74	27	78	60	22	21	30	30	37	56	74	173	1501
Aeromonas hydrophila	2172	540	284	172	134	91	139	77	41	45	33	90	127	324	708	2344
Aggregatibacter actinomycetemcomitans	701	200	118	88	66	120	24	40	21	17	31	40	55	90	208	1324
Anaplasma phagocytophilum	504	207	100	84	67	64	71	53	63	30	19	26	43	12	39	761
Bacillus amyloliquefaciens	1111	594	327	235	125	74	85	43	50	46	64	100	60	81	147	2889
Bacillus cereus	3779	895	427	270	230	191	179	156	104	96	106	107	134	152	341	3226
Bacillus pumilus	1350	367	215	135	108	66	91	60	60	42	34	48	62	107	473	2490
Bacillus subtilis	1487	401	171	185	135	66	60	53	38	49	53	68	99	205	718	2389

Bacillus thuringiensis	4381	907	366	290	300	165	163	98	111	127	144	127	129	238	585	3020
Bacteroides fragilis	4188	870	753	414	239	252	237	157	111	94	65	70	74	155	376	2461
Bifidobacterium animalis	285	140	50	16	8	6	6	7	12	5	12	18	55	69	109	1213
Bifidobacterium bifidum	860	238	92	90	32	44	23	28	51	51	24	25	26	39	112	1291
Bifidobacterium breve	941	303	162	74	61	55	85	40	51	29	31	45	47	57	126	1218
Bifidobacterium longum	1583	604	407	248	117	88	57	63	37	25	53	35	43	118	102	1134
Bordetella bronchiseptica	1295	394	177	118	110	82	50	40	44	37	33	67	49	103	270	3709
Bordetella holmesii	359	100	35	19	19	20	37	10	25	15	12	16	34	243	163	2681
Bordetella pertussis	787	68	31	32	47	40	11	18	29	65	213	53	22	47	294	2632
Borrelia burgdorferi	181	51	5	7	5	1	3	2	4	5	7	5	7	33	71	687
Brachyspira hyodysenteriae	262	159	67	67	18	52	8	11	13	7	20	9	25	51	150	2011
Brucella abortus	322	209	73	48	13	8	7	10	6	8	40	50	90	132	308	2307
Brucella melitensis	330	85	51	67	88	34	7	9	28	43	37	30	36	148	605	2068
Brucella ovis	204	10	0	6	3	0	4	2	1	1	0	1	2	4	451	2591
Brucella suis	106	43	45	114	320	40	35	40	65	79	56	47	30	93	161	2421
Buchnera aphidicola	254	31	1	8	9	6	3	7	7	7	27	61	105	99	91	156
Burkholderia mallei	1675	351	239	103	39	21	22	22	33	69	147	140	291	234	539	2955
Burkholderia pseudomallei	2930	442	182	109	76	110	107	51	39	42	38	17	38	53	195	4742
Campylobacter coli	402	167	154	82	51	99	42	29	9	15	17	34	127	117	227	1015
Campylobacter jejuni	716	184	62	47	79	59	29	24	16	13	19	46	33	71	298	1013
Chlamydia psittaci	118	41	13	7	4	12	6	11	15	4	4	2	8	10	31	901
Chlamydia trachomatis	81	16	16	13	8	12	10	5	5	2	7	11	10	10	40	803
Citrobacter freundii	3242	733	898	387	210	191	120	72	65	65	69	91	75	96	251	3229
Clostridium botulinum	2693	1069	455	1305	221	89	103	89	76	253	1122	249	55	43	236	805
Comamonas testosteroni	3272	947	457	496	309	110	140	84	100	83	77	100	119	132	179	3369
Corynebacterium diphtheriae	548	185	157	124	97	50	35	34	34	37	25	36	37	56	287	1482
Corynebacterium pseudotuberculosis	239	82	55	36	17	20	24	24	24	30	41	42	32	58	209	1616
Enterobacter aerogenes	2852	511	258	152	151	57	67	85	33	34	30	39	71	155	1305	2493
Enterobacter asburiae	2960	789	410	279	182	149	107	90	85	81	71	56	36	123	366	3085
Enterobacter cloacae	2465	753	453	234	191	118	181	87	126	88	59	51	126	85	258	2966
Enterococcus faecalis	1141	323	124	91	50	206	155	138	125	82	49	55	30	25	635	1512
Enterococcus faecium	1346	509	315	231	141	134	110	77	55	79	53	49	71	103	93	1786
Escherichia coli	2159	950	541	328	282	206	212	178	159	80	66	86	68	117	431	2780
Francisella tularensis	226	64	56	10	9	4	19	12	26	32	34	49	84	88	190	1136

Fusobacterium nucleatum	1378	377	278	228	110	130	87	49	47	54	46	68	40	63	232	1182
Gallibacterium anatis	638	284	214	120	118	120	88	51	65	52	84	58	46	43	107	1510
Gardnerella vaginalis	1143	295	153	105	71	49	56	60	15	29	97	38	29	61	562	248
Haemophilus influenzae	554	232	131	137	59	81	30	40	27	16	21	28	45	53	227	1159
Haemophilus parasuis	610	202	171	117	103	93	97	104	76	74	107	199	293	346	400	227
Klebsiella oxytoca	2507	1116	472	386	221	339	197	170	202	89	32	82	64	127	459	3483
Klebsiella pneumoniae	1643	560	304	155	105	74	97	120	141	95	73	95	78	141	587	3301
Lactobacillus casei	1517	576	322	194	127	85	67	79	50	77	71	116	177	219	633	1010
Lactobacillus delbrueckii	815	247	103	102	84	67	46	61	42	52	73	69	72	147	406	606
Lactobacillus helveticus	1113	270	199	120	62	78	44	63	68	57	63	83	58	101	247	849
Lactobacillus paracasei	1453	389	223	175	115	89	76	85	56	79	73	91	144	224	562	1222
Lactobacillus plantarum	1573	401	148	128	105	144	60	75	40	53	55	51	60	155	428	1684
Lactobacillus rhamnosus	1510	273	176	90	96	81	73	78	50	82	62	77	140	349	767	806
Lactococcus lactis	1508	554	274	184	131	164	122	59	68	46	46	57	82	179	428	992
Legionella pneumophila	762	408	196	159	107	61	65	80	55	40	52	45	44	140	342	2014
Leptospira borgpetersenii	1032	833	282	119	108	115	99	76	145	135	193	94	99	197	153	2399
Leptospira interrogans	1707	514	250	158	153	146	116	91	103	96	140	50	56	102	266	2765
Leptospira kirschneri	1174	355	179	148	126	68	61	71	54	75	72	66	83	97	224	2830
Leptospira santarosai	2091	607	302	189	145	165	142	173	97	72	106	67	50	59	301	2481
Listeria monocytogenes	689	237	93	95	73	41	56	30	28	50	52	15	33	115	201	2118
Mannheimia haemolytica	620	102	74	97	48	54	34	42	74	87	125	88	73	93	403	1429
Methanosarcina mazei	688	225	155	91	105	71	47	33	38	93	70	48	42	60	119	2268
Mycobacterium abscessus	392	215	217	137	76	210	97	82	273	47	139	59	41	43	121	3773
Mycobacterium africanum	116	12	3	1	1	1	1	1	0	2	0	1	1	9	151	3342
Mycobacterium avium	2798	482	149	168	47	63	125	35	54	78	104	122	268	515	1003	1688
Mycobacterium bovis	439	144	78	42	78	22	25	38	33	42	54	41	45	102	480	2674
Mycobacterium tuberculosis	579	109	57	61	42	41	40	31	49	42	37	29	30	249	1028	2021
Neisseria gonorrhoeae	539	202	92	67	63	57	65	49	40	47	98	53	93	106	201	1352
Neisseria meningitidis	676	151	167	73	80	40	35	35	41	41	46	49	37	47	163	1317
Oenococcus oeni	436	120	109	44	39	67	41	32	28	29	51	68	170	357	408	431
Pasteurella multocida	716	205	120	93	138	61	21	12	13	17	20	10	23	59	293	1481
Pectobacterium carotovorum	2372	492	243	138	121	111	144	59	81	72	62	43	86	201	699	2300
Prochlorococcus marinus	2131	1382	855	174	84	161	497	20	190	47	33	27	37	52	47	891
Propionibacterium acnes	499	186	101	87	36	40	27	14	20	19	33	34	59	73	186	1771
Pseudomonas aeruginosa	2033	722	324	167	132	90	53	35	51	42	42	73	112	239	759	3887

Pseudomonas fluorescens	5976	1455	765	650	416	295	339	264	238	164	187	223	140	149	448	2614
Pseudomonas putida	4710	1138	632	382	236	210	143	118	123	123	112	98	100	204	640	2818
Pseudomonas stutzeri	4073	923	421	209	170	171	143	89	69	70	80	167	227	252	515	2096
Pseudomonas syringae	7354	874	407	225	272	201	149	139	150	102	75	68	111	290	906	2403
Ralstonia solanacearum	2587	806	299	215	162	153	103	103	220	256	700	93	61	116	403	1977
Salmonella enterica	2520	830	376	182	202	188	121	105	72	78	66	113	214	255	407	2576
Serratia marcescens	2802	583	240	177	146	154	128	101	116	79	73	57	74	179	691	2804
Shigella flexneri	2821	424	227	117	79	59	79	55	61	99	144	120	166	193	435	2476
Staphylococcus aureus	632	266	189	110	71	68	36	50	20	42	66	56	36	57	168	1903
Staphylococcus epidermidis	766	319	133	125	73	83	60	25	11	20	17	24	31	95	360	1463
Streptococcus agalactiae	680	222	121	148	98	39	43	36	39	24	23	37	26	45	171	1402
Streptococcus equi	456	224	77	64	18	33	4	6	8	17	25	178	34	32	143	1369
Streptococcus mitis	2141	478	223	147	108	97	68	55	43	33	36	39	55	118	331	773
Streptococcus mutans	514	191	94	66	25	66	34	16	41	27	33	24	29	66	261	1273
Streptococcus pneumoniae	640	309	116	100	70	71	49	51	42	43	25	45	52	60	189	1298
Streptococcus sanguinis	1310	352	144	155	89	76	70	49	51	51	44	27	31	100	458	1212
Streptococcus suis	879	322	171	99	48	38	22	39	69	54	24	39	69	61	186	1271
Streptococcus thermophilus	643	210	92	63	41	44	51	54	46	57	63	60	59	68	177	1083
Treponema denticola	517	431	344	182	86	90	70	60	68	44	51	33	76	136	74	1729
Vibrio cholerae	1831	284	216	145	89	101	37	33	28	36	41	92	69	98	384	2533
Vibrio parahaemolyticus	2568	443	150	152	60	138	148	62	62	35	25	33	86	106	458	3294
Vibrio vulnificus	2220	556	210	282	217	166	122	102	81	59	81	145	127	210	519	2585
Xanthomonas axonopodis	2787	546	242	136	250	116	147	88	90	78	71	66	86	212	736	2275
Xanthomonas campestris	3243	490	266	221	246	215	122	123	109	159	103	63	49	80	371	2465
Xanthomonas citri	321	20	6	43	112	1	1	1	8	14	41	7	7	69	149	3793
Xanthomonas euvesicatoria	543	71	58	28	7	10	16	11	24	25	24	23	124	52	129	3712
Xanthomonas perforans	685	81	146	27	24	55	16	10	28	15	27	12	18	149	357	3459
Xylella fastidiosa	1569	322	228	107	84	66	67	50	32	32	28	47	68	107	261	1254
Yersinia enterocolitica	2116	517	258	158	96	152	103	121	74	87	28	42	41	86	336	2772
Yersinia frederiksenii	2372	968	646	242	184	211	110	71	108	82	63	92	122	112	201	2846
Yersinia pestis	814	185	96	59	52	41	47	41	35	76	45	45	103	281	292	2699
Yersinia pseudotuberculosis	1200	539	196	123	64	76	59	53	29	22	43	46	53	99	216	3082
